# Partitioning defective 6 homolog alpha (PARD6A) promotes epithelial–mesenchymal transition via integrin β1-ILK-SNAIL1 pathway in ovarian cancer

**DOI:** 10.1038/s41419-022-04756-2

**Published:** 2022-04-05

**Authors:** Ziwen Lu, Sirui Yuan, Lingling Ruan, Zhigang Tu, Hanqing Liu

**Affiliations:** 1 School of Pharmacy, Jiangsu University, Zhenjiang, Jiangsu 212013 China; 2 School of Life Sciences, Jiangsu University, Zhenjiang, Jiangsu 212013 China

**Keywords:** Ovarian cancer, Oncogenesis

## Abstract

Partitioning-defective protein 6 (Par6) family proteins have been demonstrated to be closely associated with the occurrence and development of cancers. It is well accepted that dysregulation of epithelial–mesenchymal transition (EMT) greatly contributes to carcinogenesis and metastases of ovarian cancer. So far, the roles of Par6 in EMT of ovarian cancer are not clear. Functional experiments were carried out to study the roles of PARD6A in EMT of ovarian cancer in vitro and in vivo, and EMT pathways potentially affected by PARD6A expression were screened. We found that PARD6A was significantly highly expressed in tissues of ovarian cancer patients in III-IV stages, poorly differentiated or with lymphatic metastases versus I-II stages, moderately or well differentiated, or without lymphatic metastases, respectively. PARD6A knockdown suppressed EMT of SKOV3 and A2780 cells in vitro and ovarian cancer metastasis in vivo, while overexpression of PARD6A promoted EMT in HO8910 and OVCAR8 cells. It was indicated that PARD6A affected EMT of ovarian cancer cells through SNAIL1 signaling pathway and subsequently modulated the expression of VIMENTIN and E-cadherin, which was further confirmed by knockdown and overexpression of SNAIL1 experiments. PARD6A was also demonstrated to regulate expression of SNAIL1 by modulating integrin β1 and ILK proteins, specifically it was shown that the transcription of SNAIL1 was regulated by ILK in this study. In addition, expression of ILK in ovarian cancer tissues was demonstrated to be correlated with tumor stages and lymphatic metastases clinically. In this study, we identified a novel role of PARD6A as an inducer of cell migration and invasion, which is likely to play an important role in metastasis of ovarian cancer. The molecular pathways of EMT mediated by PARD6A-Integrin β1-ILK-SNAIL1 and finally implemented by E-cadherin and VIMENTIN may provide a novel strategy for drug development for ovarian cancer therapy in the near future.

## Introduction

Partitioning-defective protein 6 (Par6) family, one family of the par proteins in mammals, contains three members, namely partitioning defective 6 homolog alpha (PARD6A), partitioning defective 6 homolog beta (PARD6B), and partitioning defective 6 homolog gamma (PARD6G), which enconde Par6α, β, and γ, respectively [1, 2]. By forming the ‘PAR complex’ with Par3, aPKC, and Cdc42 [3, 4], Par6 proteins are important for asymmetric cell division, apical-basal and anterior-posterior polarity, directional migration, adhesion, etc [2, 5–7].

Recent studies have demonstrated that Par6 family is closely correlated with the occurrence and development of a variety of cancers [5, 8]. For example, Par6 has been demonstrated to promote glioma cell proliferation and colony formation, and its expression is relevant to the malignancy and poor prognosis in glioma patients [8]. Nolan et al. reported that Par6 was overexpressed in breast cancers and promoted cell proliferation [9]. Emerging evidence suggests that activation of epithelial–mesenchymal transition (EMT) plays a pivotal role in many pathological processes, such as tissue fibrosis, tumor invasiveness, and metastasis [10, 11]. Par6 has been implicated in EMT of several different cancers. In breast, prostate, and lung cancer cells, TGF-β-dependent EMT requires the phosphorylation of Par6 [3, 12–14]. In contrast, some other studies indicate the complex roles of Par6 in the EMT processes. For instance, Zhang et al. reported that Shp2 enhanced EMT of prostate cancer by attenuating the PAR complex [15]. Zhou et al. reported that downregulation of PAR complex led to EMT of lung adenocarcinoma cells [16].

As the most lethal gynecological cancer, ovarian cancer has an overall 5-year survival rate only at 38–40% in the world [17, 18]. At the time of diagnosis, ovarian cancer has often spread to pelvic organs due to the invasion and metastasis of ovarian cancer cells through peritoneal fluid or ascites [19]. It is well accepted that EMT greatly contributes to progression, metastasis, transcriptional regulation, and chemotherapy resistance of ovarian cancer cells with clinical relevance [20–24].

So far, the roles of PARD6A in EMT of cancers originating from epithelial cells are unclear. More importantly, there haven’t been any studies focusing on the effects of PARD6A on EMT in ovarian cancer which is particularly prone to migrate and invade. In the current study, starting from the cellular functions of PARD6A, we studied the roles of PARD6A in EMT of ovarian cancer cells and the underlying mechanisms regulating these functions.

## Results

### PARD6A clinically correlates with ovarian cancer

**Fig. 1:**
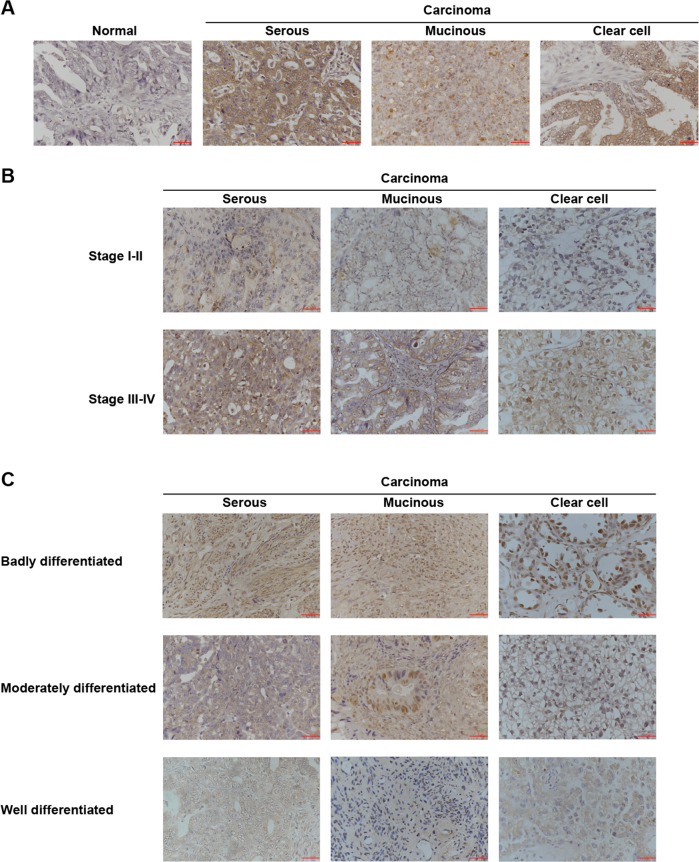
PARD6A potentially correlates with ovarian cancer clinically. **A** Representative images of immunohistochemistry detections of protein levels of PAR6α in serous, mucinous, and clear cell subtypes of ovarian cancer tissues versus normal ovarian epithial tissues. **B** Representative images of immunohistochemistry detections of protein levels of PAR6α in Stages I-II versus Stages III-IV of serous, mucinous, and clear cell subtypes of ovarian cancer tissues. **C** Representative images of immunohistochemistry detections of protein levels of PAR6α in poorly, moderately, and well differentiated ovarian cancer tissues in serous, mucinous, and clear cell subtypes. Scale bar = 50 µm.

To evaluate whether PARD6A is clinically associated with ovarian cancers (serous, mucinous, and clear cell ovarian cancers), expression of PARD6A protein PAR6α were studied in patients’ tissues versus normal ovarian epithial tissues using immunohistochemistry assays (Fig. 1A, Table 1). PAR6α expression was significantly higher in patients’ tissues with ovarian cancers compared to normal ovarian epithial tissues (Table 1, *P* = 0.022), but the expression among the three subtypes were not significantly different. PAR6α exhibited significantly higher expression in stage III-IV than its expression in Stage I-II (Fig. 1B, Table 1, *P* = 0.042). Additionally, PAR6α expression was significantly higher in badly differentiated ovarian cancer tissues than moderately or well differentiated cacer tissues (Fig. 1C, Table 1, *P* = 0.021). Furthermore, PAR6α expression was observed at a significantly higher level in tissues with metastases (*P* = 0.041). All these results indicate that PARD6A clinically correlates well with metastases of ovarian cancer.

**Table 1:** Clinical and pathological characteristics of patients with ovarian cancers in the current study.

Clinicopathological characteristics	Number of patients	% of total (*n*_total_ = 76)	Number of patients with PAR6α expression			*P* value
			weak	moderate	strong	
Age						
<50	27	35.53	7	12	8	0.398
≥50	49	64.47	11	22	16	
Tumor stage						
I-II	31	40.79	10	15	6	0.042
III-IV	45	59.21	8	19	18	
Lymphatic metastasis						
Absent	44	57.89	13	21	10	0.041
Present	32	42.11	5	13	14	
Differentiation						
Badly differentiated/Grade 3 or undifferentiated/Grade 4	42	55.26	10	19	13	0.021
Moderately differentiated/Grade 2	11	14.47	0	5	6	
Well differentiated/Grade 1	23	30.26	8	10	5	
Histological type						
Serous	49	64.47	11	18	20	0.229
Mucinous	17	22.37	4	11	2	
Clear cell	10	13.16	3	5	2	

Tissues	Number of patients	% of total (*n*_total_ = 82)	Number of patients with PAR6α expression			*P* value
			weak	moderate	strong	
Normal	6	7.32	4	2	0	0.022
Ovarian cancer	76	92.68	18	34	24	

### PARD6A knockdown suppresses EMT of ovarian cancer cells in vitro and in vivo

**Fig. 2:**
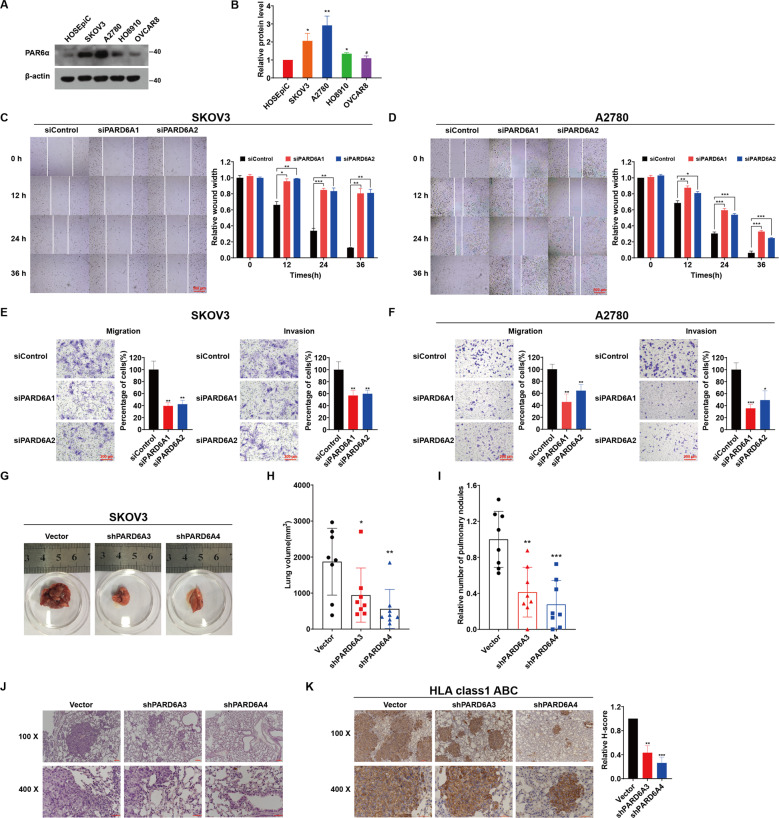
PARD6A knockdown suppresses EMT of ovarian cancer cells in vitro and in vivo. **A** Protein levels of PAR6α in ovarian cancer cell lines (SKOV3, HO8910, OVCAR8 and A2780) and normal ovarian surface epithelial cell line HOSEpiC. Above cells were collected and protein lysates were subjected to immunoblot analysis with the PAR6α antibody. **B** Densitometric analysis of the immunoblots (**A**) was conducted using ImageJ to quantify PAR6α levels relative to β-actin in ovarian cancer cell lines. Representative pictures and statistical analysis of wound-healing assays on SKOV3 (**C**) and A2780 cells (**D**), which were silenced with siPARD6As, at the indicated time. Scale bar = 500 µm. Representative pictures of transwell migration and invasion assays and statistical analysis of transwell migration and invasion assays on SKOV3 (**E**) and A2780 cells (**F**) silenced by siPARD6As. Scale bar = 200 µm. **G** Representative images of lung metastases of ovarian cancer cells in mice. **H** Statistics of volumes of lungs of each group of mice at the indicated time. **I** Pulmonary nodules of each group of mice. **J** Representative images of H&E staining of paraffin sections of mouse lung tissues in each group. Scale bar = 100 µm (×100) and scale bar = 50 µm (×400). **K** Immunohistochemical detection of HLA class1 ABC in mouse lung tissues. Data are expressed as the H-Score, considering both staining intensity and the percentage of positively staining cells. Scale bar = 100 µm (×100) and scale bar = 50 µm (×400). Data shown in this figure are the mean values (±SD) from three independent experiments. Statistically significant differences with *P* < 0.05 were considered significant (*P < 0.05; **P < 0.01; ***P < 0.001).

To study the roles of PARD6A in ovarian cancer cells, protein expression of PARD6A in ovarian cancer cells and normal ovarian surface epithelial cell line HOSEpiC were first evaluated. Western blotting results showed that expression of PAR6α protein was significantly higher in the ovarian cancer cell lines, SKOV3, A2780, HO8910, and OVCAR8 (Fig. 2A, B). Expression of PAR6α in SKOV3 and A2780 cell lines were relatively higher, and thus they were later selected for knockdown experiments. And HO8910 and OVCAR8 cell lines were chosen for overexpression experiments due to the lower expression of PAR6α in them. These results confirmed that PAR6α is highly expressed in many ovarian cancer cells and indicated that PARD6A gene would likely play an important role in ovarian cancer.

To investigate the roles of PARD6A in ovarian cancer cell lines, a few siRNAs were synthesized and screened (Fig. S1A) and a couple of them (siPARD6A1 and siPARD6A1) were used to silence PARD6A (Table S1). Both siPARD6A1 and siPARD6A2 efficiently suppressed the mRNA (Fig. S1B) and protein expression levels (Fig. S1C) of PARD6A in both SKOV3 and A2780 cell lines. But PARD6A knockdown didn’t significantly affect proliferation of SKOV3 and A2780 cells (Fig. S2A, B). However, PARD6A knockdown significantly suppressed migration of SKOV3 (Fig. 2C) and A2780 cells (Fig. 2D) manifested by the results of wound-healing assays. PARD6A knockdown significantly suppressed migration of SKOV3 and A2780 cells after 12 h. In addition, results from transwell assays confirmed that migration and invasion of both SKOV3 (Fig. 2E) and A2780 cells (Fig. 2F) were significantly inhibited by PARD6A silencing.

To further evaluate the roles of PARD6A in vivo, xenograft mouse models of metastasis were built with injection of PARD6A manipulated SKOV3 cells in the tail veins, and lung metastasis status from different groups was evaluated (Fig. 2G). Firstly, the silencing efficiency of PARD6A (Fig. S3A, B) and capabilities of these shRNAs to suppress EMT (Fig. S3C, D) was verified, and shPARD6A3 and shPARD6A4 were chosen for in vivo experiments since they were capable to suppress the expression of PARD6A and inhibit EMT in vitro. Not surprisingly, cell viability was not significantly affected by shPARD6As (Fig. S3E). Consistent with results of in vitro experiments, PARD6A knockdown significantly suppressed metastases of ovarian cancer cells in vivo. Results showed that the volumes of lungs and numbers of pulmonary nodules were significantly decreased after PARD6A silenced with shPARD6A3 and shPARD6A4 (Fig. 2H, I). H&E staining of pulmonary tissues showed that there were obvious metastases in lungs of the control group, showing dense intercellular space, while shPARD6A groups had much milder lung metastases with much larger intercellular space (Fig. 2J). To confirm that the lung metastases were in fact human ovarian cancer cells rather than mouse tissues, immunohistochemistry assays using the anti-HLA class 1 ABC antibody were applied to pulmonary tissues. HLA class 1 ABC is not present in mouse cells, so this antibody can specifically label nodules formed by migrated SKOV3 cells in mouse lungs. The staining results showed that H-scores were significantly lower in shPARD6A groups than the control group (*P* < 0.01 for shPARD6A3 and *P* < 0.001 for shPARD6A4) (Fig. 2K). These results confirmed that the pulmonary nodules of the mice were formed by SKOV3 ovarian cancer cells. In sum, knockdown of PARD6A gene suppressed EMT of ovarian cancer cells in vitro and in vivo.

### Overexpression of PARD6A promotes EMT of ovarian cancer cells in vitro

**Fig. 3:**
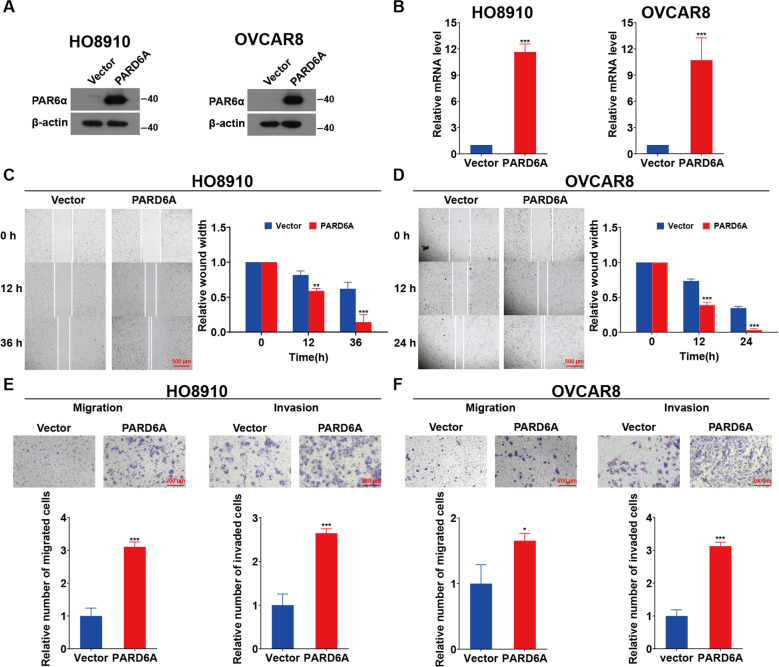
Overexpression of PARD6A promotes EMT of ovarian cancer cells in vitro. **A**, **B** PARD6A was successfully overexpressed in HO8910 and OVCAR8 cells. **A** Protein lysates of HO8910 and OVCAR8 cells with PARD6A overexpression were subjected to immunobloting with the PAR6α antibody and β-actin was used as an internal control. **B** Relative mRNA expression of PARD6A in HO8910 and OVCAR8 cells with PARD6A overexpression assessed by qRT-PCR. Representative images and statistical analysis of wound-healing assays on HO8910 (**C**) and OVCAR8 cells (**D**), which were overexpressed with PARD6A, at the indicated time. Scale bar = 500 µm. Representative images and statistical analysis of transwell migration and invasion assays on HO8910 (**E**) and OVCAR8 cells (**F**), which were overexpressed with PARD6A. Scale bar = 200 µm. Data shown in **B**–**E** are the mean values (±SD) from three independent experiments. Statistically significant differences with *P* < 0.05 were considered significant (*P < 0.05; **P < 0.01; ***P < 0.001).

To further confirm that PARD6A plays an important role in migration and invasion of ovarian cancer cells, the constructs were built to overexpress PARD6A in ovarian cancer cell lines. Both western blot and qRT-PCR results demonstrated that PARD6A was successfully overexpressed (Fig. 3A, B). Again, MTT assays showed that there was no significant difference on cell viability with PARD6A overexpression (Fig. S2C, D), not surprising since PARD6A silencing didn’t significantly affect cell viability either. The wound healing and transwell assays showed that cells with PARD6A overexpression migrated significantly faster in both HO8910 and OVCAR8 cell lines (Fig. 3C–F). In addition, PARD6A overexpression also promoted cell invasion significantly in both cell lines (Fig. 3E, F). Together with earlier results, it was indicated that PARD6A promotes migration and invasion of ovarian cancer cells.

### EMT-associated signaling pathways affected by PARD6A expression

**Fig. 4:**
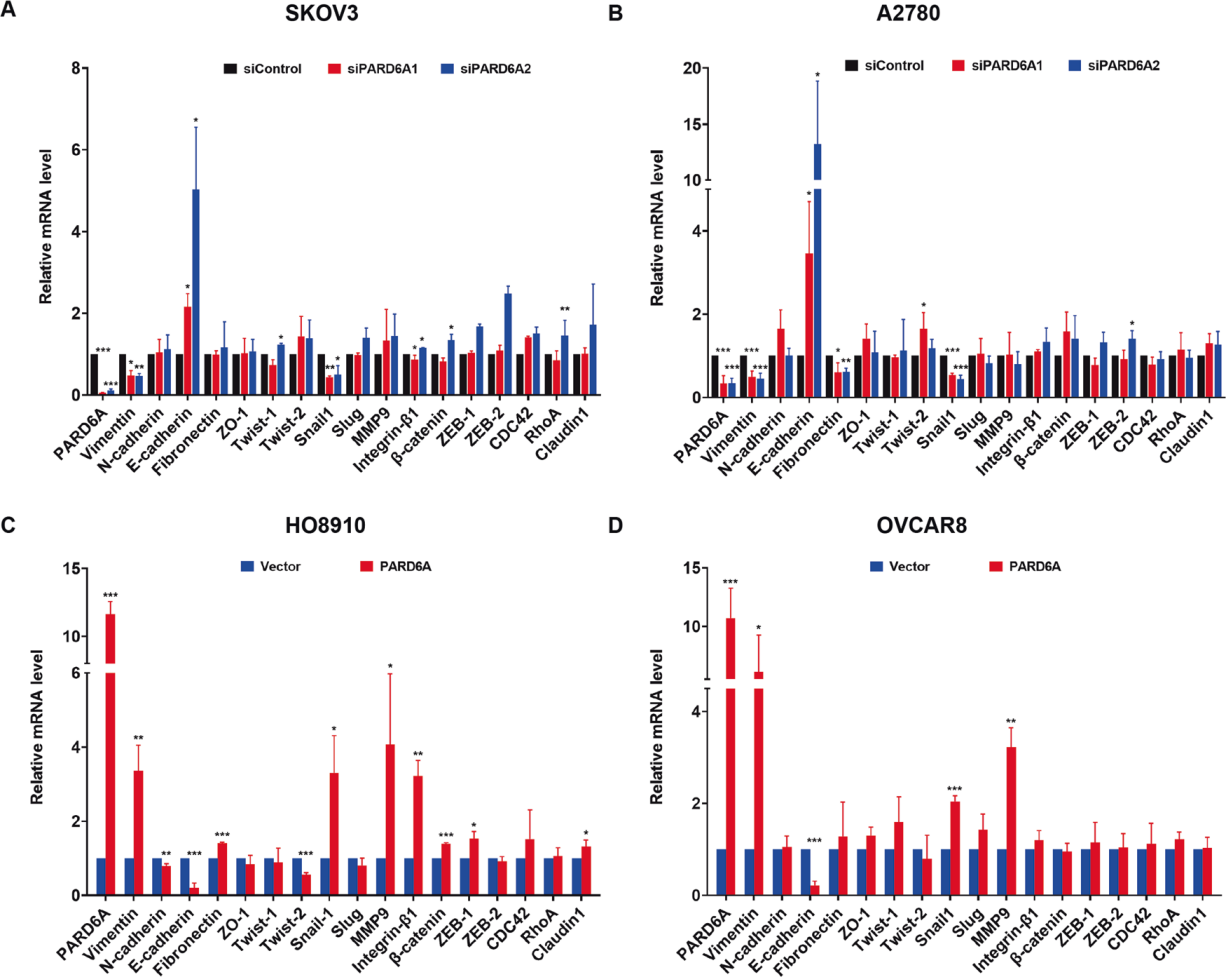
mRNA expression of EMT-associated genes potentially affected by PARD6A expression. mRNA expression of EMT-associated genes in SKOV3 (**A**) and A2780 cells (**B**) with PARD6A silenced. mRNA expression of EMT-associated genes in HO8910 (**C**) and OVCAR8 cells (**D**) with PARD6A overexpressed. Data shown are the mean values (±SD) from three independent experiments and relative values of mRNA expression of the genes when expression of PARD6A manipulated versus the expression of them when transfected with siControls. Statistically significant differences with *P* < 0.05 were considered significant (*P < 0.05; **P < 0.01; ***P < 0.001).

To further investigate the molecular mechanisms how PARD6A expression affects migration and invasion of ovarian cancer cells, we were interested in searching for signaling pathways involved in these processes. Seventeen proteins closely associated with migration and invasion were thus selected for further studies [25–39]. The results showed that the mRNA levels of SNAIL1 and VIMENTIN were significantly down-regulated, while the mRNA levels of E-cadherin were significantly up-regulated consistently in both SKOV3 and A2780 cells after PARD6A knockdown with both siRNAs, when compared to the control groups (Fig. 4A, B). mRNA levels of some other genes were significantly changed either in only one cell line or only with one of the siRNAs applied. Interestingly, the mRNA levels of SNAIL1, MMP9, and VIMENTIN were up-regulated while that of E-cadherin was down-regulated after successful overexpression of PARD6A in both HO8910 and OVCAR8 cells (Fig. 4C, D). And mRNA expression of some other genes was significantly changed only in one cell line when PARD6A overexpressed. Overall, only the mRNA expression of SNAIL1, E-cadherin, and Vimentin exhibited significant changes in the expected ways in all four tested cell lines.

**Fig. 5:**
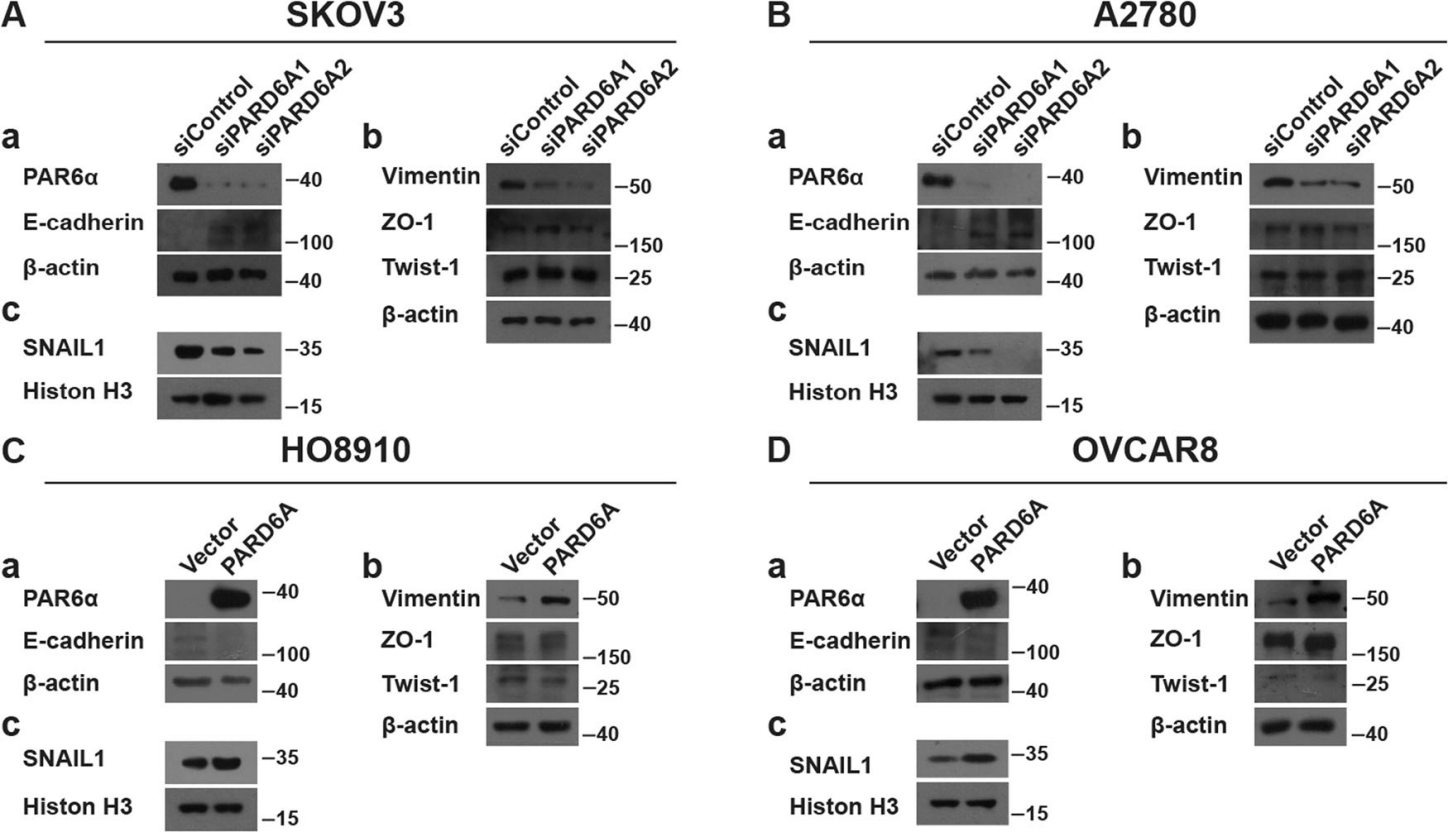
Protein levels of EMT-associated genes potentially affected by PARD6A expression. Representative western blot of EMT-associated genes in SKOV3 (**A**) and A2780 cells (**B**) with PARD6A silenced. Total protein lysates were subjected to immunoblot analysis with the indicated antibodies, except that nuclear lysates were subjected to immunoblot analysis with SNAIL1 or Histone H3 antibodies. Representative western blot of EMT-associated genes in HO8910 (**C**) and OVCAR8 cells (**D**) with PARD6A overexpressed. Total protein lysates were subjected to immunoblot analysis with the indicated antibodies, except that nuclear lysates were subjected to immunoblot analysis with SNAIL1 or Histone H3 antibodies.

The interesting proteins in these typical and atypical signaling pathways of EMT and metastasis were also explored by western blot [30, 33, 40–44]. Protein expression levels of E-cadherin, VIMENTIN, ZO-1, TWIST-1 and PAR6α were assessed in total protein lysates and protein expression levels of SNAIL1 were assessed in nuclear lysates, respectively, in multiple ovarian cancer cell lines. Consistently, protein expression levels of SNAIL1 and VIMENTIN were down-regulated and that of E-cadherin was up-regulated when PARD6A was knocked down in SKOV3 cells (Fig. 5A). Similarly, protein expression levels of these proteins changed in the same direction in A2780 cells when PARD6A was silenced (Fig. 5B). As expected, protein expression levels of SNAIL1 and VIMENTIN were up-regulated and that of E-cadherin was down-regulated when PARD6A was overexpressed in HO8910 cells (Fig. 5C). Similar results were achieved in OVCAR8 cells with PARD6A overexpression (Fig. 5D). At the same time, the protein levels of ZO-1 and TWIST-1 didn’t change much when expression of PARD6A was changed in these cell lines (Fig. 5A–D).

SNAIL1 was initially characterized as a potent repressor of E-cadherin [30, 45], a major anti-invasive molecule in carcinomas which plays an important role in embryonic development and cancer development and metastasis. VIMENTIN is also a down-stream protein of transcription factor SNAIL1 [45]. Together with the above results, PARD6A likely affects migration and invasion of ovarian cancer cells through regulating the expression of SNAIL1.

### PARD6A affects EMT of ovarian cancer cells through SNAIL1 signaling pathways

**Fig. 6:**
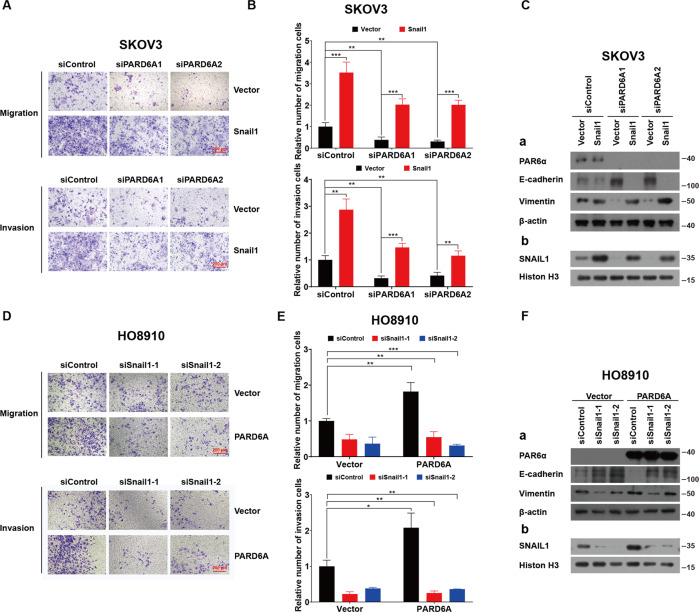
PARD6A affects EMT of ovarian cancer cells through SNAIL1 signaling pathways. **A**, **B** Overexpression of SNAIL1 reversed the effects of PARD6A silencing on EMT in SKOV3 cells. SKOV3 cells were first transfected with siPARD6As for 24 h, and then transfected with SNAIL1 overexpression plasmids. Then the cells were incubated for 6 h, and replaced with fresh medium and incubated for 24 h before experiments. Representative images (**A**) and statistical analysis (**B**) of transwell migration and invasion assays were shown here. Scale bar = 200 µm. **C** SNAIL1 overexpression reversed the change of protein levels of VIMENTIN and E-cadherin by PARD6A silencing in SKOV3 cells. Total protein lysates were subjected to immunoblot analysis with the indicated antibodies, except that nuclear lysates were subjected to immunoblot analysis with SNAIL1 or Histone H3 antibodies. **D**, **E** Silencing of SNAIL1 reversed the effects of PARD6A overexpression on EMT in HO8910 cells. HO8910 cells were first infected with lentivirus to overexpress PARD6A, and then treated with siSNAIL1s to silence SNAIL1. Representative images (**D**) and statistical analysis (**E**) of transwell migration and invasion assays were shown here. Scale bar = 200 µm. **F** Silencing of SNAIL1 reversed the change of protein levels of VIMENTIN and E-cadherin by PARD6A overexpression in HO8910 cells. Total protein lysates were subjected to immunoblot analysis with the indicated antibodies, except that nuclear lysates were subjected to immunoblot analysis with SNAIL1 or Histone H3 antibodies. Data shown in **B** and **E** are the mean values (±SD) from three independent experiments. Statistically significant differences with *P* < 0.05 were considered significant (*P < 0.05; **P < 0.01; ***P < 0.001).

To test our hypothesis, SNAIL1 overexpression constructs were first successfully built (Fig. S4A, B). PARD6A were first silenced with siRNAs in SKOV3 cells, and then SNAIL1 was overexpressed. Migrated and invaded cells were significantly decreased by PARD6A silencing, but then were increased with SNAIL1 overexpression (Fig. 6A, B). Western blot results showed that the expression of PAR6α was successfully suppressed with siRNAs and SNAIL1 overexpression didn’t affect its expression (Fig. 6C). As expected, SNAIL1 overexpression down-regulated the expression of E-cadherin and enhanced the expression of VIMENTIN in SKOV3 cells with PARD6A knocked down. Similar results were also reached in A2780 cells (Fig. S4C–E). After siSNAILs were tested to be able to silence SNAIL1 in HO8910 cells (Fig. S4F), PARD6A was first overexpressed and then SNAIL1 were silenced. Results of transwell migration and invasion assays showed that after SNAIL1 silencing, the percentages of migrated and invaded cells significantly decreased (Fig. 6D, E). Results of western blots demonstrated that PAR6α was successfully overexpressed and SNAIL1 was successfully knocked down with siRNAs (Fig. 6F). The increase of the expression of VIMENTIN after PARD6A overexpression was reversed due to SNAIL1 silencing. In addition, the decrease of the expression of E-cadherin because of PARD6A overexpression was also reversed by SNAIL1 silencing. In summary, our results indicated that PARD6A affected EMT of ovarian cancer cells through SNAIL1 signaling pathways, which subsequently modulated the expression of VIMENTIN and E-cadherin.

### Expression of PARD6A regulates the protein levels of integrin β1 and ILK

**Fig. 7:**
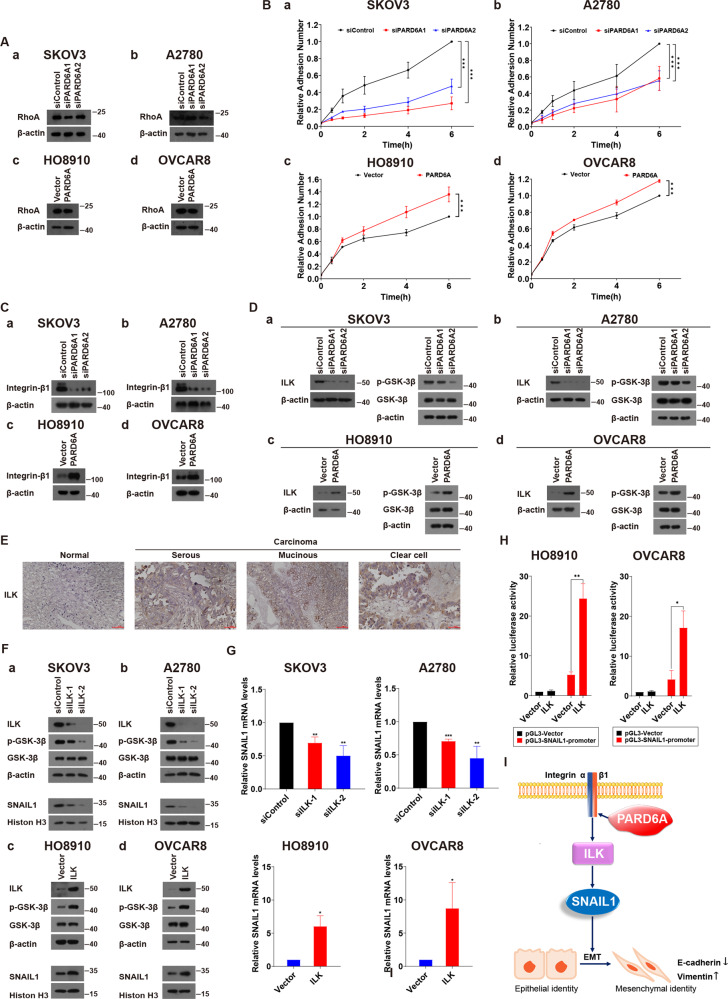
Expression of PARD6A regulates the protein levels of integrin β1 and ILK. **A** Expression of PARD6A didn’t significantly affect protein levels of RhoA. **a**, **b** Protein levels of RhoA in SKOV3 (**a**) and A2780 cells (**b**) with PARD6A silenced. **c**, **d** Protein levels of RhoA in HO8910 (**c**) and OVAR8 cells (**d**) with PARD6A overexpressed. **B** Expression of PARD6A significantly affected cell adhesion. **a**, **b** Relative cell adhesion values of SKOV3 (**a**) and A2780 cells (**b**) with PARD6A silenced. **c**, **d** Relative cell adhesion values of HO8910 (**c**) and OVAR8 cells (**d**) with PARD6A overexpressed. Data shown are the mean values (±SD) from three independent experiments. Statistically significant differences with *P* < 0.05 were considered significant (***P < 0.001). **C** Expression of PARD6A regulates the protein levels of integrin β1. **a**, **b** Protein levels of Integrin β1 in SKOV3 (**a**) and A2780 cells (**b**) with PARD6A silenced. **c**, **d** Protein levels of Integrin β1 in HO8910 (**c**) and OVAR8 cells (**d**) with PARD6A overexpressed. **D** Expression of PARD6A regulates the protein levels of integrin β1. **a**, **b** Protein levels of ILK, p-GSK3β, and GSK3β in SKOV3 (**a**) and A2780 cells (**b**) with PARD6A silenced. **c**, **d** Protein levels of ILK, p-GSK3β, and GSK3β in HO8910 (**c**) and OVAR8 cells (**d**) with PARD6A overexpressed. **E** Representative images of immunohistochemistry detections of protein levels of ILK in serous, mucinous, and clear cell subtypes of ovarian cancer tissues versus normal ovarian epithial tissues. Scale bar = 50 µm. **F**, **H** Expression of ILK regulates expression of SNAIL1. **F** Protein levels of SNAIL1 in nuclear lysates and protein levels of ILK, p-GSK3β, and GSK3β in total protein lysates of SKOV3 and A2780 cells with ILK silenced and those levels of the indicated proteins in HO8910 and OVAR8 cells with ILK overexpression, respectively. **G** Relative mRNA expression of SNAIL1 in SKOV3 and A2780 cells with ILK silenced and that of SNAIL1 in HO8910 and OVCAR8 cells with ILK overexpression assessed by qRT-PCR. **H** Relative luciferase activity of pGL3-SNAIL1-promotor with/without ILK overexpression. **I** Scheme of the molecular pathways of EMT mediated by PAR6α-Integrin β1-ILK-SNAIL1 and finally implemented by E-cadherin and VIMENTIN.

After obtaining the above results, we wanted to further explore the mechanisms by which the expression of PARD6A affected SNAIL1 expression. Previous studies have shown that the decrease of RhoA protein plays a crucial role in the process of TGF-β-Par6 mediated EMT in breast cancer cells [3]. Therefore, the protein levels of RhoA was first tested. As shown in Fig. 7A, the protein levels of RhoA did not change significantly in any of four types of ovarian cancer cells no matter in which PARD6A was overexpressed or silenced. These results made us to search for other potential mechanisms. After many trials without desired results, we accidentally found that the numbers of adhesion cells with PARD6A knockdown decreased significantly, and on the contrary, the numbers of adhesion cells with PARD6A overexpression increased significantly (Fig. 7B). Because cell adhesion is closely associated with the expression of integrin β1 and integrins was also demonstrated to play important roles in migration and invasion of cancer cells [46, 47], the expression of integrin β1 was tested in such cells. Indeed, the protein levels of integrin β1 increased as PARD6A overexpressed, and vice versa (Fig. 7C). Since integrin-linked kinase (ILK) is a β1-integrin cytoplasmic domain interacting protein and it is also closely related to EMT, then ILK was also studied [48, 49]. It was found that the protein levels of ILK changed with Par6 expression similarly (Fig. 7D). Phosphorylation of GSK3β have been reported to be the main downstream players of ILK signaling pathways [50, 51]. Therefore, expression of p-GSK3β was used as an indication for ILK activity changes (Fig. 7D). Expression of p-GSK3β was decreased when PARD6A was silenced while that of p-GSK-3β was increased when PARD6A was overexpressed. In addition, expression of ILK in ovarian cancer tissues was much higher than that in normal ovarian epithelial tissues (Fig. 7E). ILK was significantly highly expressed in the tissues of ovarian cancer patients at the ages older than 50, in III-IV stages, or with lymphatic metastases than in the tissues of ovarian cancer patients younger than 50, I-II stages, or without lymphatic metastases, respectively (Table S2). These results indicated that PARD6A indeed regulates the expression of integrin β1 and ILK and integrin β1-ILK pathway is probably associated with ovarian cancer clinically. It has been demonstrated that the transcription of SNAIL1 can be regulated by ILK protein in certain cancers [52, 53]. Therefore, whether SNAIL1 expression is dependent on ILK expression was examined in ovarian cancer in this study (Fig. 7F–H). Results have shown that ILK expression increased SNAIL1 protein levels and silence of ILK decreased SNAIL1 protein levels in ovarian cancer cells (Fig. 7F). The mRNA expression of SNAIL1 exhibited similar patterns when ILK expression was regulated (Fig. 7G). In addition, promoter region of SNAIL1 was inserted into pGL3-reporter vectors, and we found ILK overexpression significantly increased luciferase activities in both HO8910 and OVCAR8 cells (Fig. 7H). These results suggest that SNAIL1 expression is dependent on the ILK pathway and probably on transcription levels. The above results suggested that PARD6A regulates the mRNA and protein expression of SNAIL1 by affecting the expression of integrin β1 and ILK.

## Discussion

In the present study, the protein levels of PARD6A was found up-regulated in ovarian cancer tissues, especially in serous and mucinous ovarian tumors (Fig. 1), and then the expression levels of PARD6A in a variety of ovarian cancer cell lines were compared. In order to obtain optimized experimental results, PARD6A in SKOV3 and A2780 cell lines with high endogenous expression was knocked down and PARD6A in HO8910 and OVCAR8 cell lines with low endogenous expression levels was overexpressed. By manipulating the expression of PARD6A in cells, we studied the biological functions of PARD6A in ovarian cancer cells. Several previous studies claimed that the levels of Par6 protein were positively correlated with the rates of cell proliferation [8, 9]. To our surprise, it was found that the expression of PARD6A did not significantly affect cell proliferation in any tested cell lines in this current study. Instead, our in vitro and in vivo results (Fig. 2) clearly showed that the expression of PARD6A was positively correlated with the migration and invasion characteristics of ovarian cancer cells. In addition, it was confirmed that PARD6A regulated the migration and invasion of ovarian cancer cells through regulating important molecules in EMT pathways such as SNAIL1, E-cadherin, and VIMENTIN (Figs. 4, 5).

So far, few studies have revealed the functions of phosphorylated Par6 in EMT of cancers [14]. Several studies have discussed the functions of Par complex in EMT of cancer cells [5, 54]. The earlier studies showed that the Par6α-contained complexes facilitated EMT in NSCLC [55, 56], while Zhang et al. suggested that the complex, composed of Par6α, Par3 and aPKC, had the opposite effects on prostate cancer cells [57]. For the first time, our study clearly demonstrated that PAR6A expression was significantly associated with metastases of clinical specimens and promotes the EMT process in ovarian cancer cells. This conclusion may have some potential connection with an article published by Nakamura et al. several years ago. They found that the expression of Par3 polarity protein correlates with poor prognosis in ovarian cancer [58].

SNAIL1 was known to repress transcription of E-cadherin [59] and upregulate VIMENTIN during EMT [45]. When mRNA levels of SNAIL1, E-cadherin, and VIMENTIN were demonstrated to be affected by PARD6A expression, it was indicated that PARD6A likely affects EMT of these ovarian cancer cells through regulating SNAIL1 signaling pathway, thus acting on the downstream E-cadherin and VIMENTIN. And SNAIL1 expression was shown to be associated with distant metastases [60] and higher among primary tumors and metastases than effusions in ovarian cancer [61].

To the best of our knowledge, the regulatory effects of PAR6 on Integrin β1 was discovered by us for the first time. Inhibition of Integrin β1 was previously demonstrated to decrease the malignancy of ovarian cancer cells and potentiate anticancer therapy [46], which is consistent with our results. Previous studies showed that phosphorylation of PARD6A affects EMT through RhoA signaling pathway, our further mechanism studies revealed that rather than mediating via RhoA, knockdown or overexpression of PARD6A regulates the expression of SNAIL1 through Integrin β1 and its downstream partner ILK (Fig. 7). In addition, we also found that ILK, a cytoplasmic domain interacting protein of Integrin β1, was simultaneously regulated together with Integrin β1. ILK, bound to the cytoplasmic tails of integrin β1, β2, and β3, was known to play an important role in cancer development and therapy [62]. In addition, we found ILK expression was significantly associated with metastases of clinical specimens. And the association between ILK and cell survival and deterioration of ovarian cancer has also been reported before [63–67]. Although it has been demonstrated that the transcription of SNAIL1 can be regulated by ILK protein in certain cancers [52, 53]. In this study we further examined whether SNAIL1 expression is dependent on ILK expression in ovarian cancer. We checked protein levels and mRNA expression of SNAIL1 expression when ILK was regulated. In addition, promoter region of SNAIL1 was inserted into pGL3-reporter vectors and we found ILK overexpression significantly increased luciferase activities in both HO8910 and OVCAR8 cells (Fig. 7H). These results suggest that SNAIL1 expression is dependent on the ILK pathway and probably on transcription levels. All these data further confirmed that ILK regulates SNAIL1 expression in ovarian cancer.

Therefore, our results identified a novel role for the polarity protein PAR6α as an inducer of cell migration and invasion, which is likely to play an important role during metastasis of ovarian cancer. The molecular pathways of EMT in ovarian cancer cells mediated by PARD6A-Integrin β1-ILK-SNAIL1 and finally implemented by E-cadherin and VIMENTIN also provide a novel strategy for drug development in the future (Fig. 7I).

## Materials and methods

### Cell lines and reagents

The ovarian surface epithelial cell line HOSEpiC and ovarian cancer cell lines SKOV3, HO8910, OVCAR8 (ATCC, Manassas, VA, USA) were maintained in RPMI 1640 supplemented with 10% fetal bovine serum (FBS) and penicillin/streptomycin (100 units/mL and 100 g/mL, respectively) (Thermo Fisher Scientific, Grand Island, NY, USA). The ovarian cancer cell line A2780 (ATCC) was maintained in DMEM supplemented with 10% fetal bovine serum (FBS) and penicillin/streptomycin (Thermo Fisher Scientific). All cell lines were cultured at 37 °C in a humidified, 5% CO_2_ atmosphere, and tested for mycoplasma contamination before experiments. The antibodies used in this study were listed in Table S3. 3,3-diaminobenzidine (DAB) and hematoxylin and eosin staining kits were obtained from Gene Tech (Shanghai, China). PrimeScript RT master mix and SYBR Green qRT-PCR kit were obtained from Vazyme (Nanjing, China); Bicinchoninic acid (BCA) kit was bought from Beyotime (Shanghai, China). Polyvinylidene fluoride (PVDF) microporous membrane was purchased from Millipore (Billerica, MA, USA). The vectors PMD2.G, psPAX2, pCDH-EF1-MCS-T2A-Puro, PLP1, PLP2, PLPVSVG, PLKO.1-TRC were obtained from Addgene (Cambridge, MA, USA); the vector pcDNA 3.1 was from Thermo Fisher. The siRNAs siGL2 (targeting firefly luciferase GL2), siPARD6A1, and siPARD6A2 (targeting human PARD6A) were synthesized by GenePharma (Shanghai, China) and transfected with Lipofectamine 2000 (Thermo Fisher Scientific). The specific sequences of the siRNA used in the experiment were listed in Table S1. MTT (3- [4]-2, 5-diphenyltetrazolium bromide thiazolyl blue) was purchased from Saiguo Biotech Co. (Guangzhou, China). All chemicals are purchased from Sinopharm Co. (Beijing, China) unless otherwise specified.

### Plasmid construction

To knockdown PARD6A, four short hairpin RNA (shRNA) vectors were constructed. The shRNA sequences are shown in Table S4. The shRNAs were synthesized by Sangon Biotech (Shanghai, China). Standard molecular cloning and virus packaging were described previously [68]. The lentiviral vector was PLKO.1-TRC, and the helper plasmids used in the lentiviral packaging process were PLP1, PLP2, and PLPVSVG. Lentiviruses were packaged using Lipofectamine 2000 by following the manufacturer’s instructions. Cells infected with viruses were selected using 1 μg/ml puromycin.

To overexpress PARD6A and ILK, the full-length human PARD6A and ILK cDNAs were amplified and cloned into the BamHI and EcoRI sites of pCDH-EF1-MCS-T2A-Puro. The PCR primers were listed in Table S5. Lentiviruses were packaged using Lipofectamine 2000 by following the manufacturer’s instructions. Cells infected with viruses encoding resistance to puromycin were selected in 1 μg/ml puromycin.

To overexpress SNAIL1, the full-length SNAIL1 was synthesized by GenScript (Nanjing, China) and then cloned into the BamHI and EcoRI sites of pcDNA 3.1. The SNAIL1 expression plasmids or the empty pcDNA 3.1 plasmids were transiently transfected into A2780 and SKOV3 cells using Lipofectamine 2000 according to the manufacturer’s instructions.

### Cell proliferation assay

The cells were cultured in 96-well plates with 3000 cells per well. Viabilities of cells were measured using MTT every 24 h thereafter. Each experiment was repeated at least three times independently.

### Wound-healing assay

The cells were seeded in 24-well dishes and 10-μL pipette tips were used to create scratch wounds at the bottom of the dishes. The time intervals for the photograph recording were determined according to different types of cells. The relative wound widths were calculated relative to the wound widths at 0 h of each type of cells [69]. Each experiment was repeated at least three times independently.

### Transwell assay

Cell migration and invasion assays were also performed using Transwell (Corning, NY, USA). For invasion assays, Matrigel (BD, Warwick, Rhode Island, USA) was placed at the bottoms of the chambers; whereas there was no additional matrix for migration experiments. Experiments were performed by following the manufacturer’s instructions. Each experiment was repeated at least three times independently.

### Adhesion assay

The cells were seeded in 96-well plates with 20,000 cells per well. Then plates were placed in a CO_2_ incubator at 37 °C for cell culture. At indicated times, non-adherent cells were washed away with PBS and the number of adhesion cells were calculated using MTT. Relative adhesion numbers were calculated relative to the adhesion numbers at 6 h. Each experiment was repeated at least three times independently.

### Western blot

The standard procedures for western blotting experiments were performed as previously described [70, 71]. Antibodies used in western blot and IHC experiments were listed in Table S3. Each experiment was repeated at least three times independently.

### Real time quantitative PCR (qRT-PCR)

qRT-PCR experiments were performed as previously described [70]. To examine the expression of interested genes, total RNAs were isolated from ovarian cancer cells using Trizol Reagent (Thermo Fisher Scientific). HiScript 1st strand cDNA synthesis kit and AceQ qPCR kit were from Vazyme. The primers used for each gene were listed in Table S6. Expression of β-actin was used as an internal control. Each experiment was repeated at least three times independently.

### Ovarian cancer tissues

This study was approved by the Medical Ethics Committee of Jiangsu University. All patients provided signed written informed consent. 76 ovarian cancer tissue and 6 normal ovarian tissue specimens were collected from Affiliated Hospital of Jiangsu University. All pathological subtypes of tissues were diagnosed and confirmed by pathologists at the hospital.

### Mouse model

Animal experiments were approved by the Animal Ethics Committee of Jiangsu University. 24 athymic nude mice (8 mice/group) at the age of 5–6 weeks old were acclimatized on a standard chow diet for 1 week before the experiments and randomly grouped. 2 × 10^6^ of SKOV3 ovarian cancer cells that have been successfully silenced by shPARD6A were injected into the nude mice by tail vein injection. The control group were injected with ovarian cancer cells with vectors. According to the results from preliminary experiments, nude mice were sacrificed on the 60th day after injection, respectively. The volumes of lungs were measured and the numbers of pulmonary nodules were counted. The tissues were fixed in 10% formalin.

### Tissue preparation and hematoxylin and eosin staining

Paraffin-embedded lung tissues were sectioned at 5 µm and dried on slides. Sections were deparaffinized with xylene and hydrated in ethyl alcohol. Staining was performed as follows: after hematoxylin staining for 10 min, 1% hydrochloric acid ethanol solution was used. Then the slides were eosin stained for 1 min. After the slides were dried, images were captured using Nikon Eclipse microscope (Tokyo, Japan) for morphological evaluation. Each experiment was repeated at least three times independently.

### Immunohistochemistry

Histology and immunohistochemistry experiments were performed as previously described [70, 72]. Briefly, antigen retrieval was carried out by boiling the slides in the citrate buffer solution (pH 6.0) for 10 min. Endogenous peroxidase was blocked by pre-incubation with 0.3% hydrogen peroxide for 10 min and non-specific antigens were blocked with 10% goat serum in PBS. Then tissue sections were added with primary antibody for incubation overnight in a humidified chamber at 4 °C. Sections were exposed to appropriate secondary antibodies for 20 min the second morning. Finally, tissue sections were stained with 3,3’-diaminobenzidine (DAB) solution for 3–5 min. The immunostained sections were lightly counterstained with hematoxylin followed by dehydration through a series of alcohols and xylenes. Then images were captured using Nikon Eclipse microscope and scored. Each experiment was repeated at least three times independently.

IHC staining was independently scored by two pathologists blinded to the tissue groups. The IHC scoring is based on the staining intensity and staining area of the sections. Staining intensity was scored as 0 (negative), 1 (weak), 2 (moderate), and 3 (strong). Staining area was scored as 0 (0%), 1 (1–25%), 2 (26–50%), 3 (51–75%), and 4 (76–100%), depending on the percentage of positive-stained cells. If there is a big discrepancy between two pathologists, the slide will be re-scored [70].

### Luciferase assay

The proximal promoter region of SNAIL1 (−1123 to +92) [53] was synthesized by GenScript. To obtain pGL3-SNAIL-promoter constructs, the DNA fragments were cloned into pGL3 Luciferase Reporter Vector (Promega, Madison, WI, USA) using KpnI and XhoI sites. The pGL3-derived plasmids (3 μg) were transfected into the target cells with pCR3.1 β-galactosidase plasmid (1 μg, as a control for transfection efficiency) by using lipofectamine 2000 (Thermo Fisher Scientific). After appropriate treatments, cells were lysed and assayed for luciferase and β-galactosidase activities. The luciferase activity of each group was obtained by normalizing luciferase activity with β-galactosidase activity in each group. The relative luciferase activity was obtained by normalizing the values against that of the control group.

### Statistical analysis

All Data are reported as mean ± SD, unless otherwise stated. Statistically significant differences between different groups were analyzed using Student’s *t* tests (two-tailed) or one-way analysis of variance, unless otherwise specified. *p* < 0.05 was considered statistically significant (*p < 0.05; **p < 0.01; ***p < 0.001) using GraphPad Prism version 5.00 (San Diego, California, USA) unless otherwise specified.

## Supplementary information

Supplementary tables

Supplementary Figure Legends

Figure S1

Figure S2

Figure S3

Figure S4

Supplementary materials-original western blots

Reproducibility checklist

## Data Availability

All data generated or analyzed during this study are included in this published article and its supplementary information files.
