# Supplementary material for: Partitioning defective 6 homolog alpha (PARD6A) promotes epithelial–mesenchymal transition via integrin β1-ILK-SNAIL1 pathway in ovarian cancer
Source: Cell Death Dis. 2022 Apr 5;13(4):304. doi: 10.1038/s41419-022-04756-2 (PMC8980072; doi:10.1038/s41419-022-04756-2)
Supplement: Supplementary file 1 — Supplementary tables [file 41419_2022_4756_MOESM1_ESM.doc]

**Supplementary Table 1. siRNAs used in this study.**

| **Name** | **Sense Sequence（5´-3´）** | **antisense Sequence（5´-3´）** |
| --- | --- | --- |
| siControl (siGL2) | CGUACGCGGAAUCUUUCGATT | UCGAAGUAUUAUCCGUACGTT |
| siPARD6A1 | CCAACAGCCAUAACCUCAUTT | AUGAGGUUAUGGCUGUUGGTT |
| siPARD6A2 | GGGCUUCUACAUCCGAGAUTT | AUCUCGGAUGUAGAAGCCCTT |
| siPARD6A3 | GGACCGTGCTACTTGGCTATTT | ATAGCCAAGTAGCACGTCCTT |
| siPARD6A4 | GGGCTTCTACATCCGAGATTT | ATCTCGGATGTAGAAGCCCTT |
| siPARD6A5 | CCAGGTTTCCTCAGTCATATT | TATGACTGAGGAAACCTGGTT |
| siPARD6A6 | GCCATAACCTCATTGTCACTT | GTGACAATGAGGTTATGGCTT |
| siSnial1-1 | GGACUUUGAUGAAGACCAUTT | AUGGUCUUCAUCAAAGUCCTT |
| siSnial1-2 | CACGAGGUGUGACUAACUATT | UAGUUAGUCACACCUCGUGTT |
| siILK-1 | UGAUCUUGGCCCCAAAAACAG | GUUUUUGGGGCCAAGAUCAAG |
| siILK-2 | GGAAGAGCAGGGACUUCAAUG | UUGAAGUCCCUGCUCUUCCUU |

**Supplementary Table 2. Clinical and pathological characteristics of patients with ovarian cancers in the current study.**

| **Clinicopathological characteristics** | **Number of patients** | **% of total**  **(n_total_=76)** | **Number of patients with ILK expression** | | | ***P* value** |  |
| --- | --- | --- | --- | --- | --- | --- | --- |
|  |  |  |  |  |  |  |  |
|  |  |  | **weak** | **moderate** | **strong** |  |  |
| **Age** |  | | | | |  |  |
| **<**50 | 27 | 35.53 | 1 | 11 | 15 | 0.041 |  |
| ≥ 50 | 49 | 64.47 | 13 | 17 | 19 |  |  |
| **Tumor stages** |  | | | | | 0.010 |  |
| I- II | 31 | 40.79 | 10 | 10 | 11 |  |  |
| III - IV | 45 | 59.21 | 4 | 18 | 23 |  |  |
| **Lymphatic Metastasis** |  | | | | | 0.041 |  |
| Absent | 44 | 57.89 | 12 | 17 | 15 |  |  |
| Present | 32 | 42.11 | 2 | 11 | 19 |  |  |
| **Differentiation** |  | | | | | 0.124 |  |
| Badly differentiated/Grade 3 or undifferentiated/Grade 4 | 42 | 55.26 | 4 | 16 | 22 |  |  |
| Moderately differentiated/Grade 2 | 11 | 14.47 | 1 | 7 | 3 |  |  |
| Well differentiated/Grade 1 | 23 | 30.26 | 9 | 5 | 9 |  |  |
| **Histological Type** |  | | | | | 0.344 |  |
| Serous | 49 | 64.47 | 8 | 16 | 25 |  |  |
| Mucinous | 17 | 22.37 | 4 | 8 | 5 |  |  |
| Clear cell | 10 | 13.16 | 2 | 4 | 4 |  |  |

**Supplementary Table 3. Antibodies used in this study.**

| **Primary antibody** | | | | |
| --- | --- | --- | --- | --- |
| **Targeted protein** | **Catalog No.** | **Source** | **Company** | **Application** |
| PAR6α | HPA041551 | Rabbit | Sigma-Aldrich | Immunohistochemistry, 1:100 |
| HLA Class 1ABC | ab70328 | Mouse | Abcam |
| PAR6α | sc-365323 | Mouse | Santa Cruz Biotech | Western blot, 1:1000 |
| RhoA | sc-418 | Mouse |
| E-cadherin | 3195 | Rabbit | Cell Signaling |
| GSK-3β | 12456 | Rabbit |
| Twist1 | 46702 | Rabbit |
| Integrin β1 | ab134179 | Rabbit | Abcam |
| p-GSK-3β | ab75814 | Rabbit |
| Vimentin | ab92547 | Rabbit |
| ZO-1 | 21773-1-AP | Rabbit | Proteintech |
| ILK | 12955-1-AP | Rabbit |
| SNAIL1 |  | Rabbit | homemade Immunogen corresponding to amino acids range：1-150 |
| β-actin | A1978 | Mouse | Sigma-Aldrich |
| **Secondary antibody** | | | | |
| Goat anti-Mouse IgG(H+L)-HRP | 115-035-003 | Goat | Jackson | Western blot, 1:5000 |
| Goat anti-Rabbit IgG(H+L)-HRP | 315-035-005 | Goat | Jackson | Western blot, 1:5000 |

**Supplementary Table 4. Sense and antisense strands of PARD6A shRNAs.**

| **Name** | **Sense Sequence（5´-3´）** | **antisenseSequence（5´-3´）** |
| --- | --- | --- |
| shPARD6A1 | CCGGCGTCGAGGTGAAGAGCAAATTCTCGAGAATTTGCTCTTCACCTCGACGTTTTT | AATTAAAAACGTCGAGGTGAAGAGCAAATTCTCGAGAATTTGCTCTTCACCTCGACG |
| shPARD6A2 | CCGGTCAGTCATAGACGTGGACCTACTCGAGTAGGTCCACGTCTATGACTGATTTTT | AATTAAAAATCAGTCATAGACGTGGACCTACTCGAGTAGGTCCACGTCTATGACTGA |
| shPARD6A3 | CCGGACGGACATGATGGTTGCCACTCGAGTGGCAACCATCATGTCCGTTTTTT | AATTAAAAAACGGACATGATGGTTGCCACTCGAGTGGCAACCATCATGTCCGT |
| shPARD6A4 | CCGGGCTGAGCCTGATAGTGACGATCTCGAGATCGTCACTATCAGGCTCAGCTTTTT | AATTAAAAAGCTGAGCCTGATAGTGACGATCTCGAGATCGTCACTATCAGGCTCAGC |

**Supplementary Table 5. Primers used for the construction of PARD6A and ILK overexpression plasmids.**

| **Name** | **Sense Sequence（5´-3´）** | **antisense Sequence（5´-3´）** |
| --- | --- | --- |
| PARD6A | TAGAATTCGCCACCATGGCCCGGCCGCAGAGGACT | GCGCGGATCCGAGGCTGAAGCCACTACCATCTCCT |
| ILK | GTGACCGGCGCCTACTCTAGAGCCACCATGGACGACATT | GGCCGCGGATCCTTCGAATTCCTACTTGTCCTGCATCTT |

**Supplementary Table 6. Primers for qRT-PCR detection used in this study.**

| **Gene** | **Primer1（5’-3’）** | **Primer2（5’-3’）** |
| --- | --- | --- |
| CDC42 | AGTGGGYGCCTGAGATAACT | CTCCAGGGCAGCCAATATTG |
| CDH1 | AAAACAGCAAAGGGCTTGGA | GCAGTGTCTCTCCAAATCCGATA |
| CDH2 | CGATCCCAATGCCCTCAATG | TGCCTTCCATGTCTGTAGCT |
| Claudin1 | CTGGGAGGTGCCCTACTTTG | ACACGTAGTCTTTCCCGCTG |
| Fibronectin | GGACATGCATTGCCTACTCG | GAATCCTGGCATTGGTCGAC |
| Integrin-β1 | GCCGCGCGGAAAAGATGAAT | GAATTTGTGCACCACCCACAA |
| MMP9 | TCTATGGTCCTCGCCCTGAA | CATCGTCCACCGGACTCAAA |
| PARD6A | ACGATGACAGCAGTGACCTG | AGAGGCTGAAGCCACTACCA |
| RhoA | ACAGCTGGGCAGGAAGATTA | CGCCTTGTGTGCTCATCATT |
| slug | ACGCCTCCAAAAAGCCAAAC | ACTCACTCGCCCCAAAGATG |
| Snail1 | CCCCAATCGGAAGCCTAACT | GGACAGAGTCCCAGATGAGC |
| Twist1 | GGAGTCCGCAGTCTTACGAG | GCTTGAGGGTCTGAATCTTGC |
| Twist2 | CGCAAGTGGAATTGGGATGC | CGATGTCACTGCTGTCCCTT |
| Vimentin | GGACCAGCTAACCAACGACA | AAGGTCAAGACGTGCCAGAG |
| ZEB1 | GGTGTACCAGAGGATGACCTG | GCTTGAGGGTCTGAATCTTGC |
| ZEB2 | AGTGTGCCCAACCATGAGTC | GGTCTGGATCGTGGCTTCTG |
| ZO-1 | AGCCATTCCCGAAGGAGTTG | CAGCTCCACAGGCTTCAGG |
| β-catentin | GAGGAAGGTCTGAGGAGCAGC | TGTCCAACTCCATCAAATCAGCTTG |
| ILK | TGGATCACTCCACAGTCCTCA | TTACATTGATCCGTGCCCCC |
| β-actin | AGAGCTACGAGCTGCCTGAC | AGCACTGTGTTGGCGTACAG |
