# Supplementary material for: Partitioning defective 6 homolog alpha (PARD6A) promotes epithelial–mesenchymal transition via integrin β1-ILK-SNAIL1 pathway in ovarian cancer
Source: Cell Death Dis. 2022 Apr 5;13(4):304. doi: 10.1038/s41419-022-04756-2 (PMC8980072; doi:10.1038/s41419-022-04756-2)
Supplement: Supplementary file 2 — Supplementary Figure Legends [file 41419_2022_4756_MOESM2_ESM.docx]

**Supplementary Figure Legends**

**Supplementary Figure 1. PARD6A was successfully silenced in SKOV3 and A2780 cells with siRNAs. A**. Relative mRNA expression of PARD6A after siPARD6A silencing with six siPARD6As in A2780 cells. **B**. Relative mRNA expression of PARD6A after siPARD6A silencing with six siPARD6As in SKOV3 and A2780 cells. β-actin was used as the internal control. Relative mRNA expression was calculated relative to the expression when transfected with siControl. Data shown are the mean values (±SD) from three independent experiments. Statistically significant differences with P<0.05 were considered significant (**P<0.01; ***P<0.001). **C**. Protein levels of PAR6α after siPARD6A silencing in SKOV3 and A2780 cells. The protein lysates were subjected to immunoblotting using PAR6α antibody.

**Supplement Figure 2.** **Silencing or overexpression of PARD6A shows no significant effects on the viabilities of ovarian cancer cells. A-B**. PARD6A silencing did not significantly affect the viabilities of SKOV3 (**A**) and A2780 cells (**B**). **C-D**. PARD6A overexpresssion did not significantly affect the viabilities of HO8910 (**C**) and OVCAR8 cells (**D**). Cell viabilities were assessed using MTT assays. Data shown are the mean values (±SD) from three independent experiments.

**Supplement Figure 3. PARD6A was successfully silenced in SKOV3 cells with shRNAs and PARD6A knockdown suppresses EMT. A**. Protein levels of PAR6α after shPARD6A silencing in SKOV3 cells. The protein lysates were subjected to immunoblotting using PAR6α antibody. **B**. Densitometric analysis of the immunoblots (**A**) was conducted using ImageJ to quantify PAR6α levels relative to β-actin in SKOV3 cells. **C-D**. Statistical analysis of transwell migration and invasion assays in SKOV3 cells silenced by shPARD6As. **E**. No significant changes in cell viabilities after shPARD6A silencing in SKOV3 cells. Data shown in **B-E** are the mean values (±SD) from three independent experiments. Statistically significant differences with P<0.05 were considered significant (*P<0.05; **P<0.01; ***P<0.001).

**Supplement Figure 4. PARD6A expression was successfully manipulated and expression of PARD6A affects EMT of A2780 cells through SNAIL1 signaling pathways. A-B**. SNAIL1 was successfully overexpressed in SKOV3 (**A**) and A2780 cells (**B**). SKOV3 and A2780 cells were transfected with SNAIL1 overexpressing plasmids, and total RNAs were extracted for qRT-PCR experiments. **C-D**. Overexpression of SNAIL1 reversed the effects of PARD6A silencing on EMT in A2780 cells. A2780 cells were first transfected with siPARD6As for 24 hours, and then transfected with SNAIL1 overexpression plasmids. Then the cells were incubated for 6 hours, and replaced with fresh medium and incubated for 24 hours before experiments. Representative images (**C**) and statistical analysis (**D**) of transwell migration and invasion assays were shown here. Scale bar=200 µm. **E**. SNAIL1 overexpression reversed the change of protein levels of Vimentin and E-cadherin by PARD6A silencing in A2780 cells. **F**. SNAIL1 was successfully silenced in HO8910 cells by siSNAILs. Data shown in this figure are the mean values (±SD) from three independent experiments. Statistically significant differences with P<0.05 were considered significant (*P<0.05; **P<0.01; ***P<0.001).
