# Supplementary figures and images for: Partitioning defective 6 homolog alpha (PARD6A) promotes epithelial–mesenchymal transition via integrin β1-ILK-SNAIL1 pathway in ovarian cancer
Source: Cell Death Dis. 2022 Apr 5;13(4):304. doi: 10.1038/s41419-022-04756-2 (PMC8980072; doi:10.1038/s41419-022-04756-2)

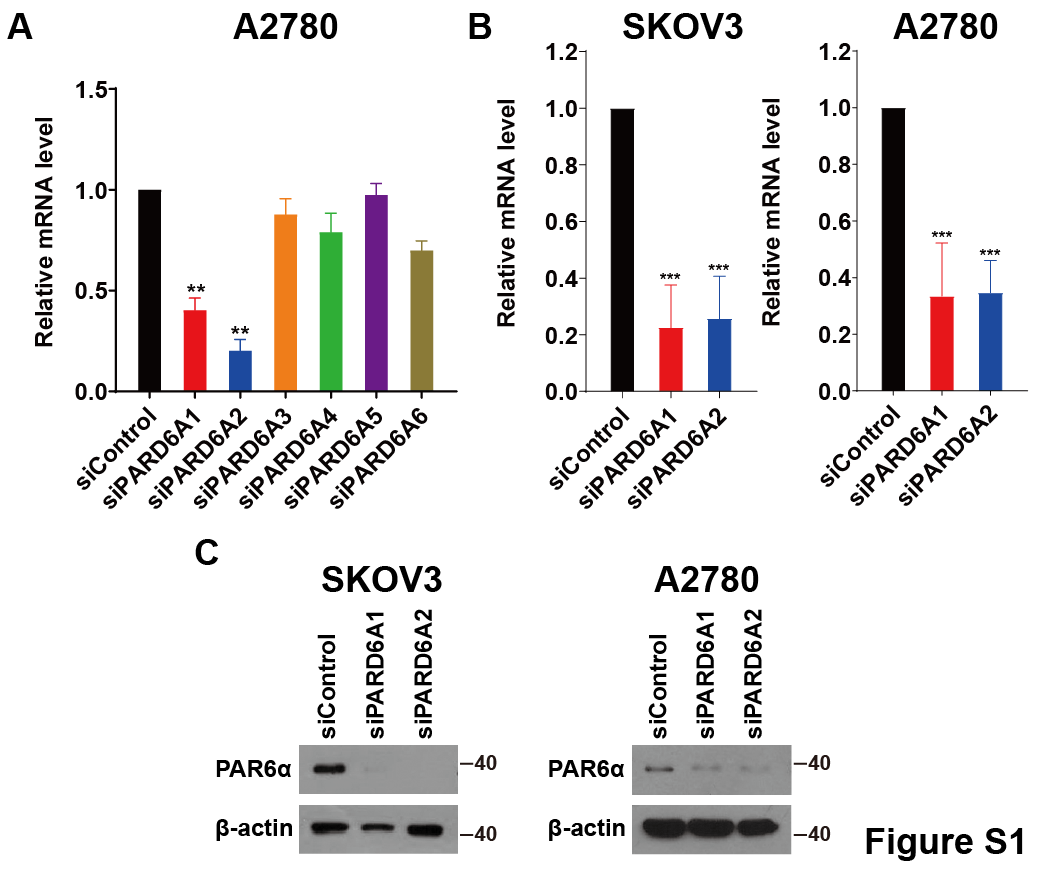

Supplement: Supplementary file 3 — Figure S1 [file 41419_2022_4756_MOESM3_ESM.tif]

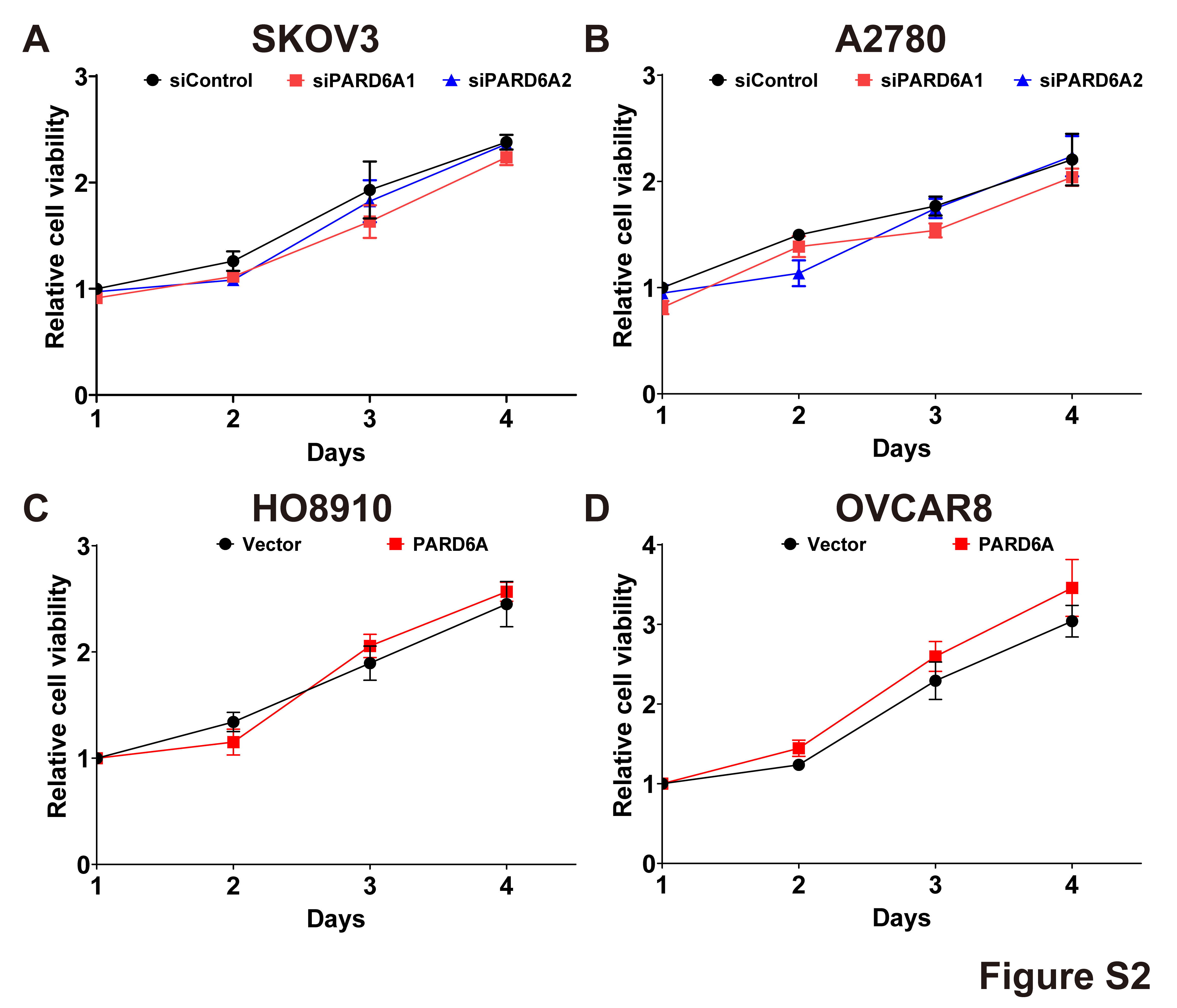

Supplement: Supplementary file 4 — Figure S2 [file 41419_2022_4756_MOESM4_ESM.tif]

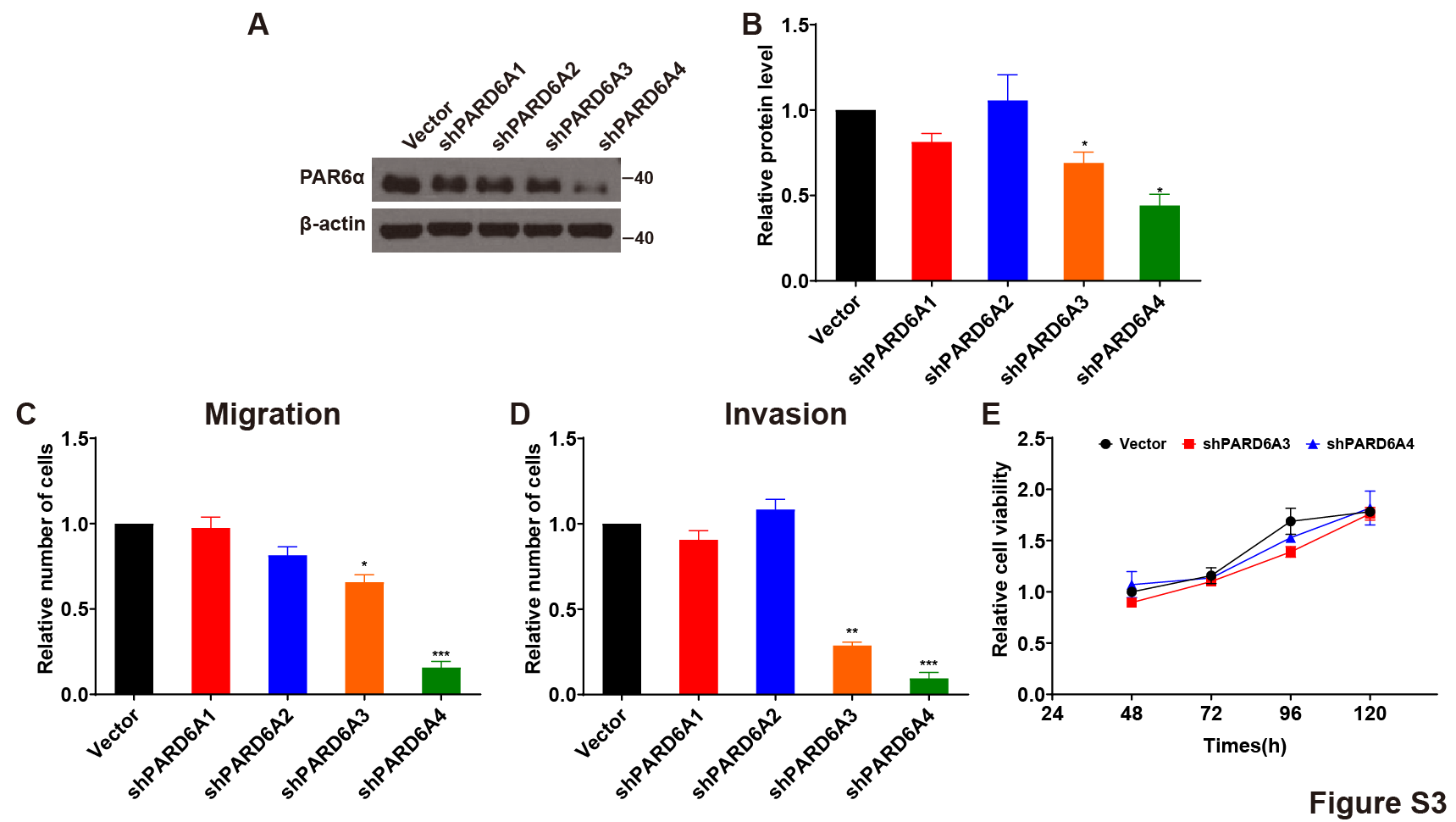

Supplement: Supplementary file 5 — Figure S3 [file 41419_2022_4756_MOESM5_ESM.tif]

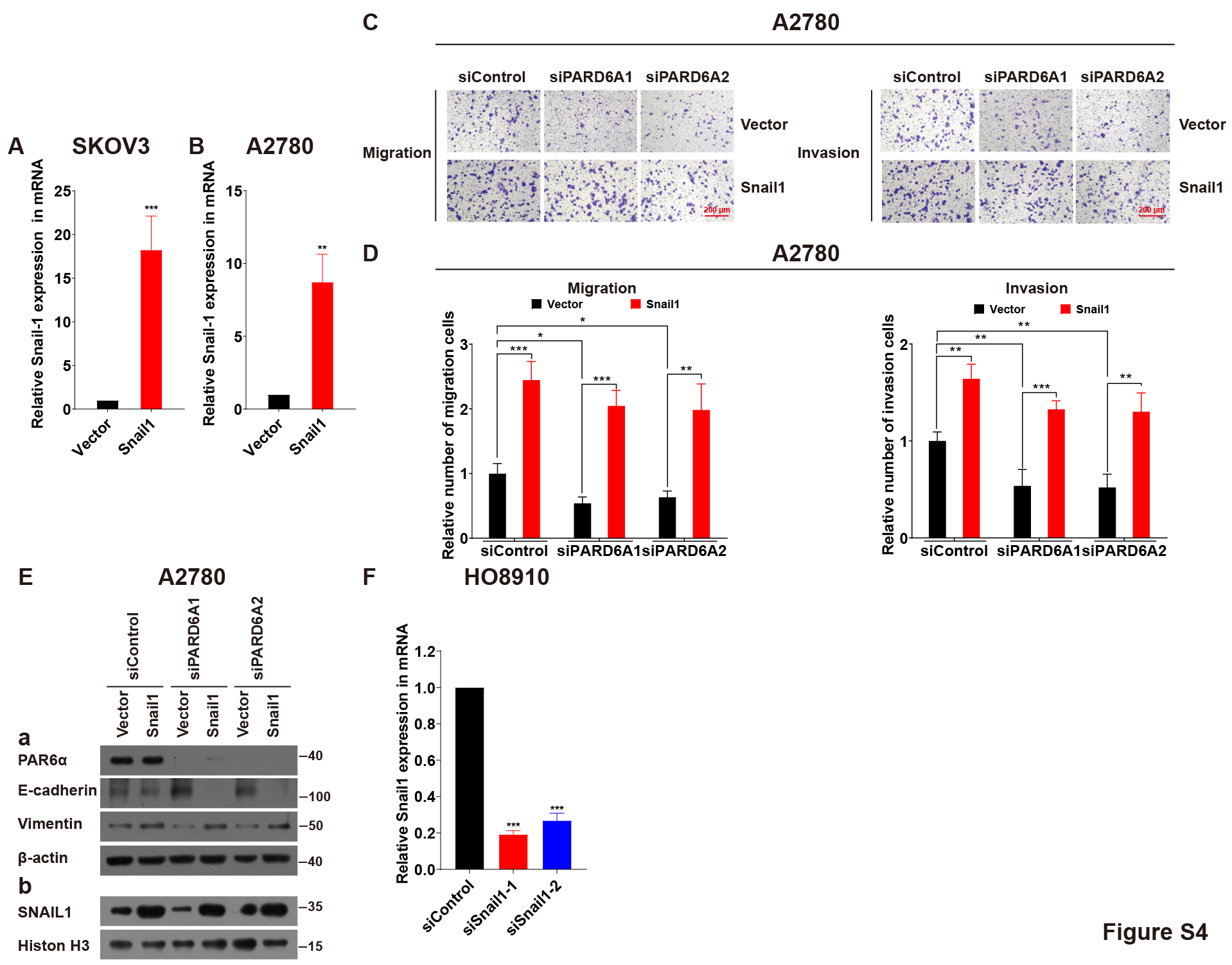

Supplement: Supplementary file 6 — Figure S4 [file 41419_2022_4756_MOESM6_ESM.tif]
