# Supplementary material for: Partitioning defective 6 homolog alpha (PARD6A) promotes epithelial–mesenchymal transition via integrin β1-ILK-SNAIL1 pathway in ovarian cancer
Source: Cell Death Dis. 2022 Apr 5;13(4):304. doi: 10.1038/s41419-022-04756-2 (PMC8980072; doi:10.1038/s41419-022-04756-2)

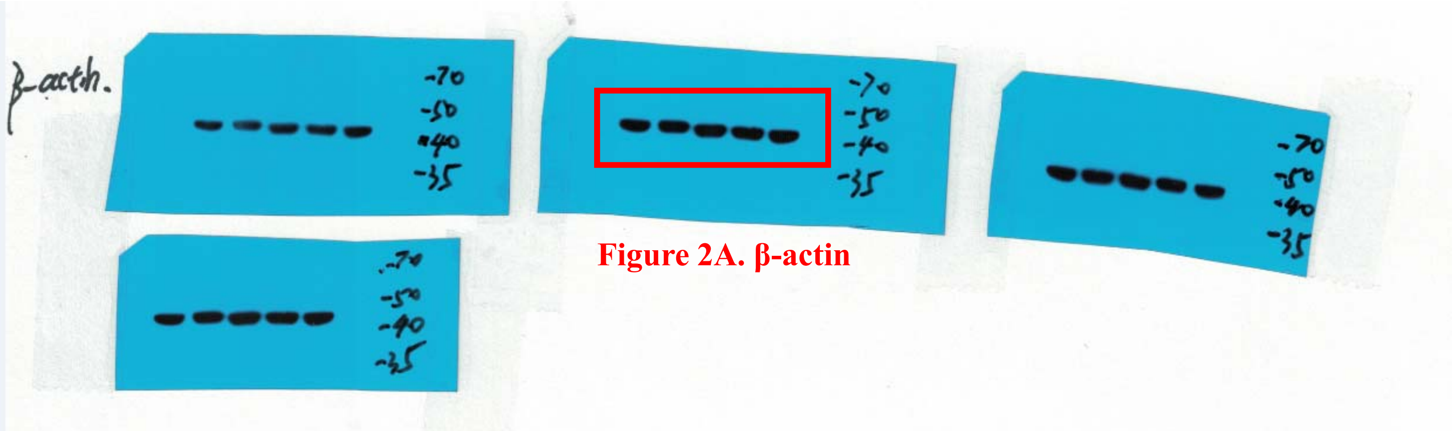

Figure 2A. β-actin

Figure 2A. PARD6A

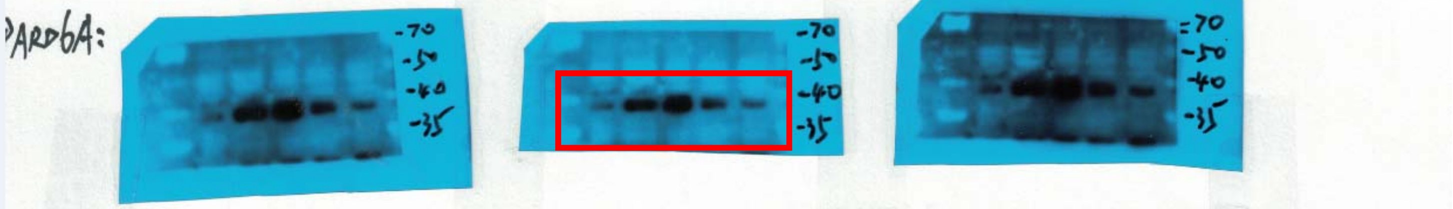

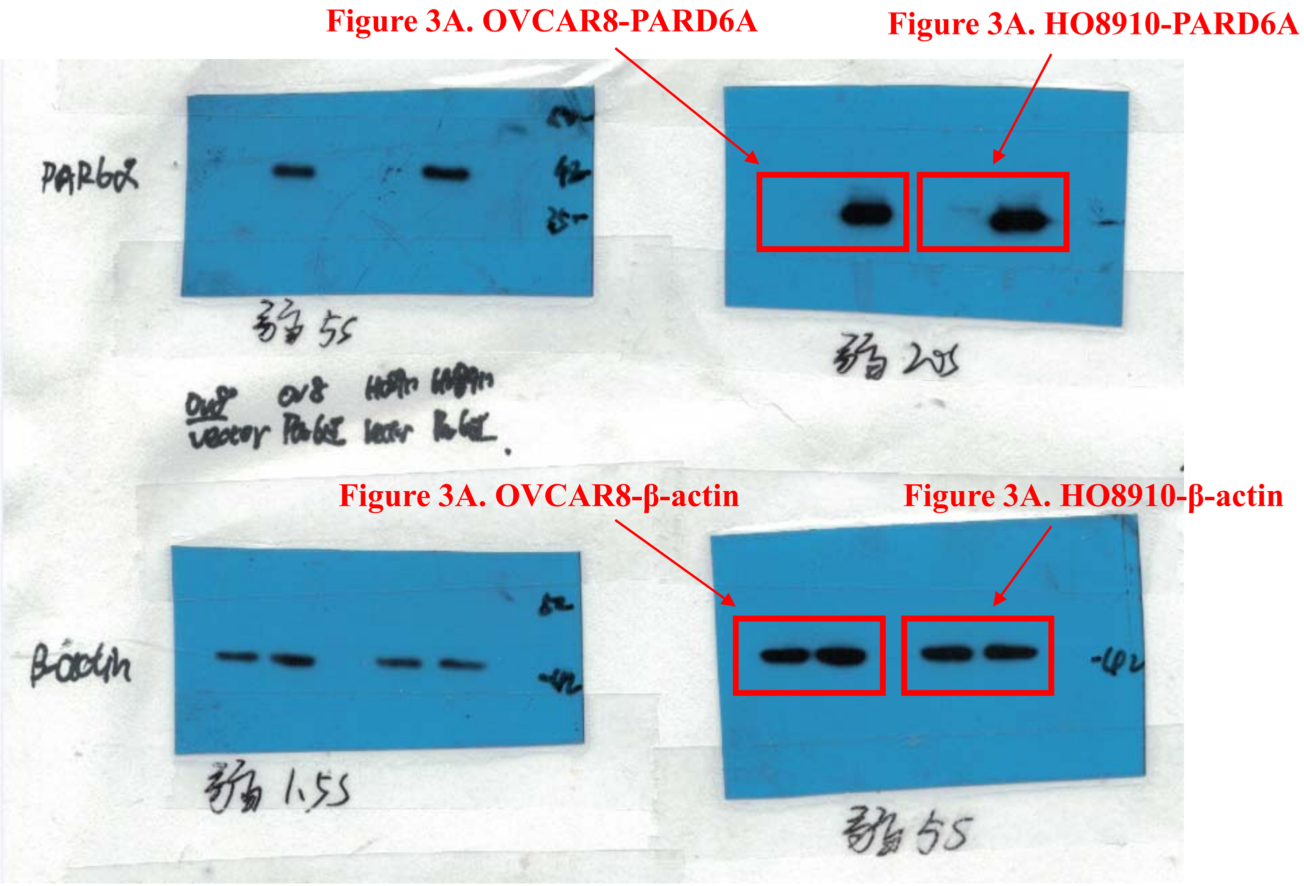
Figure 3A. OVCAR8-PARD6A
Figure 3A. HO8910-PARD6A
Figure 3A. OVCAR8-β-actin
Figure 3A. HO8910-β-actin

Figure 5A. SKOV3

PARD6A

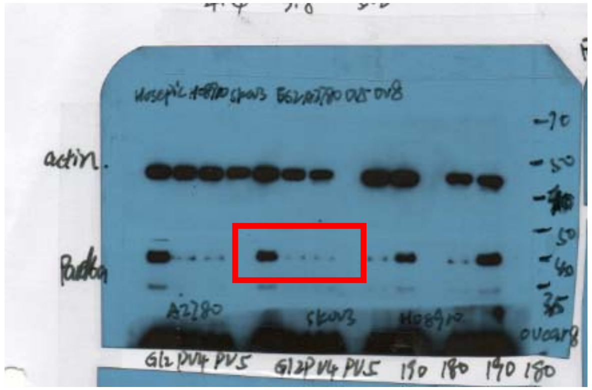

E-cadherin

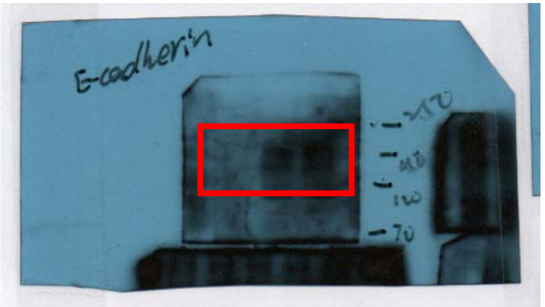

β-actin

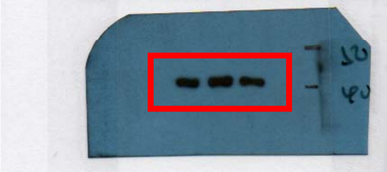

ZO-1

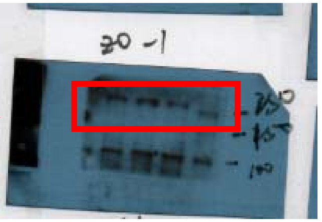

Vimentin

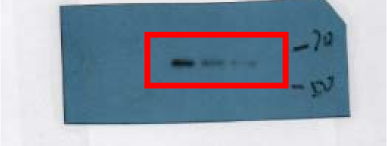

Twist-1

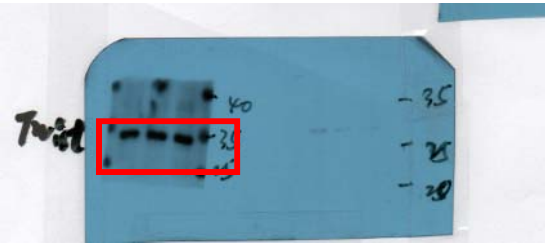

β-actin

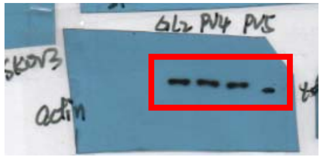

Figure 5B. A2780

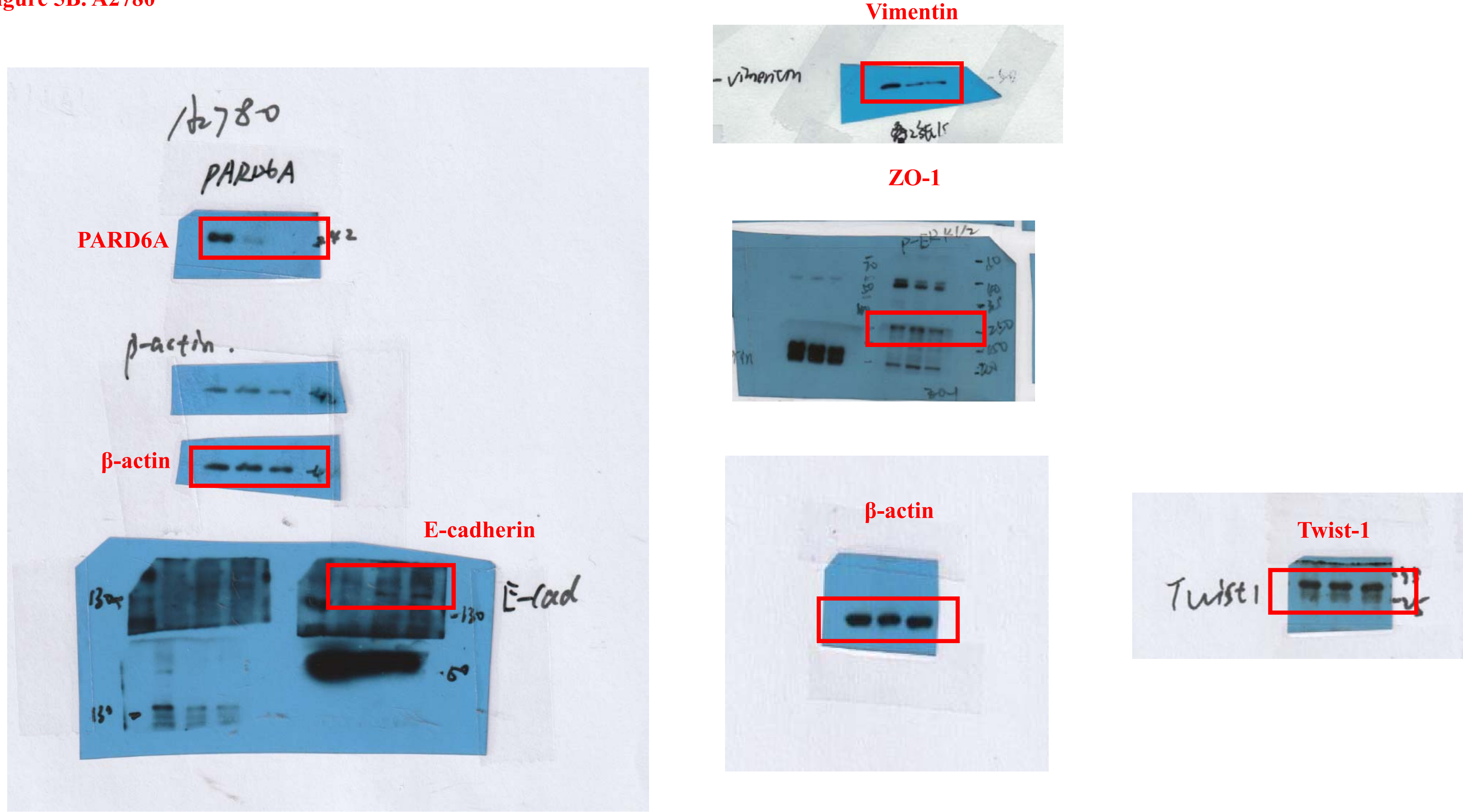

Figure 5C. HO8910

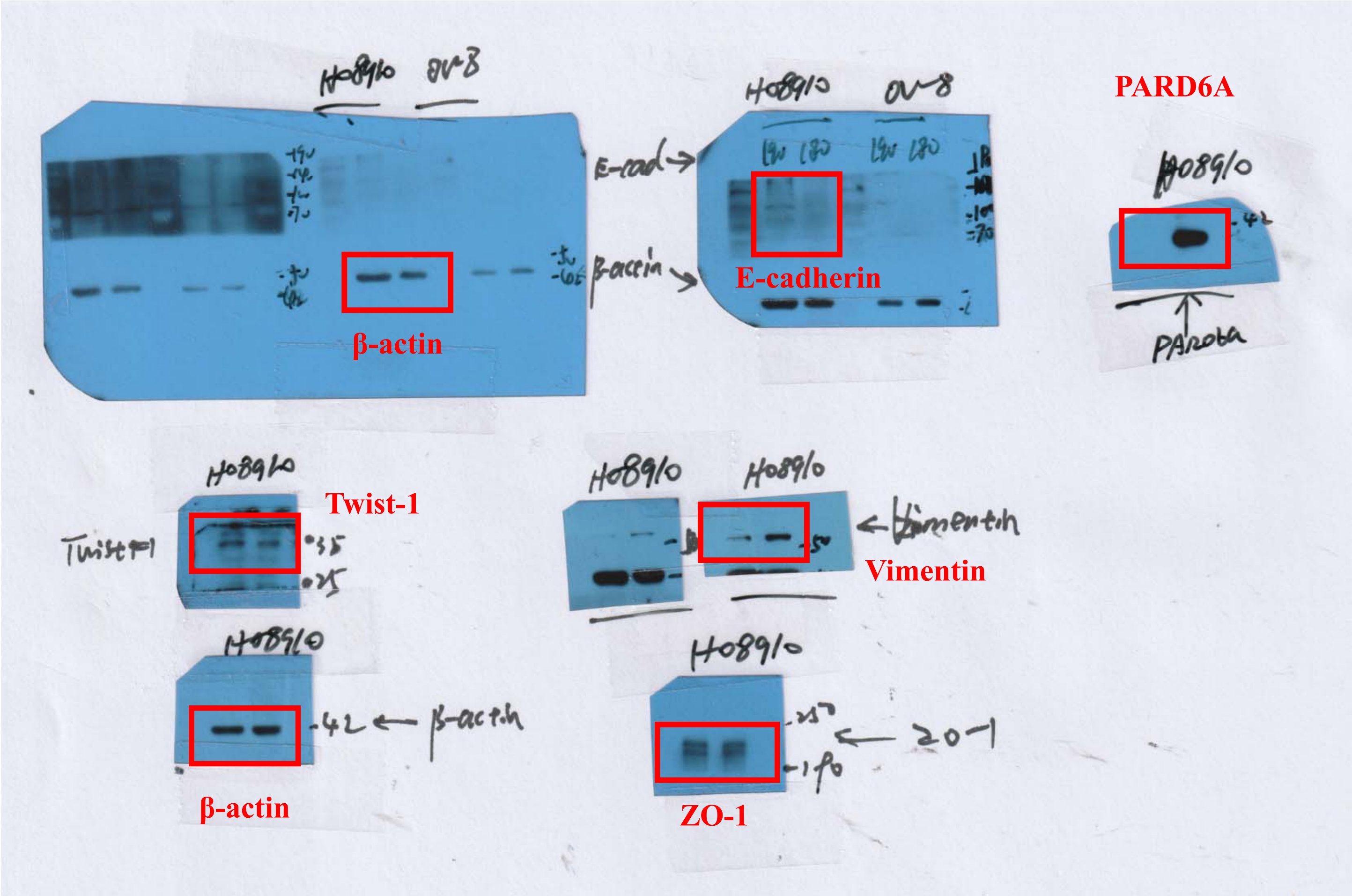

Figure 5D. OVCAR8

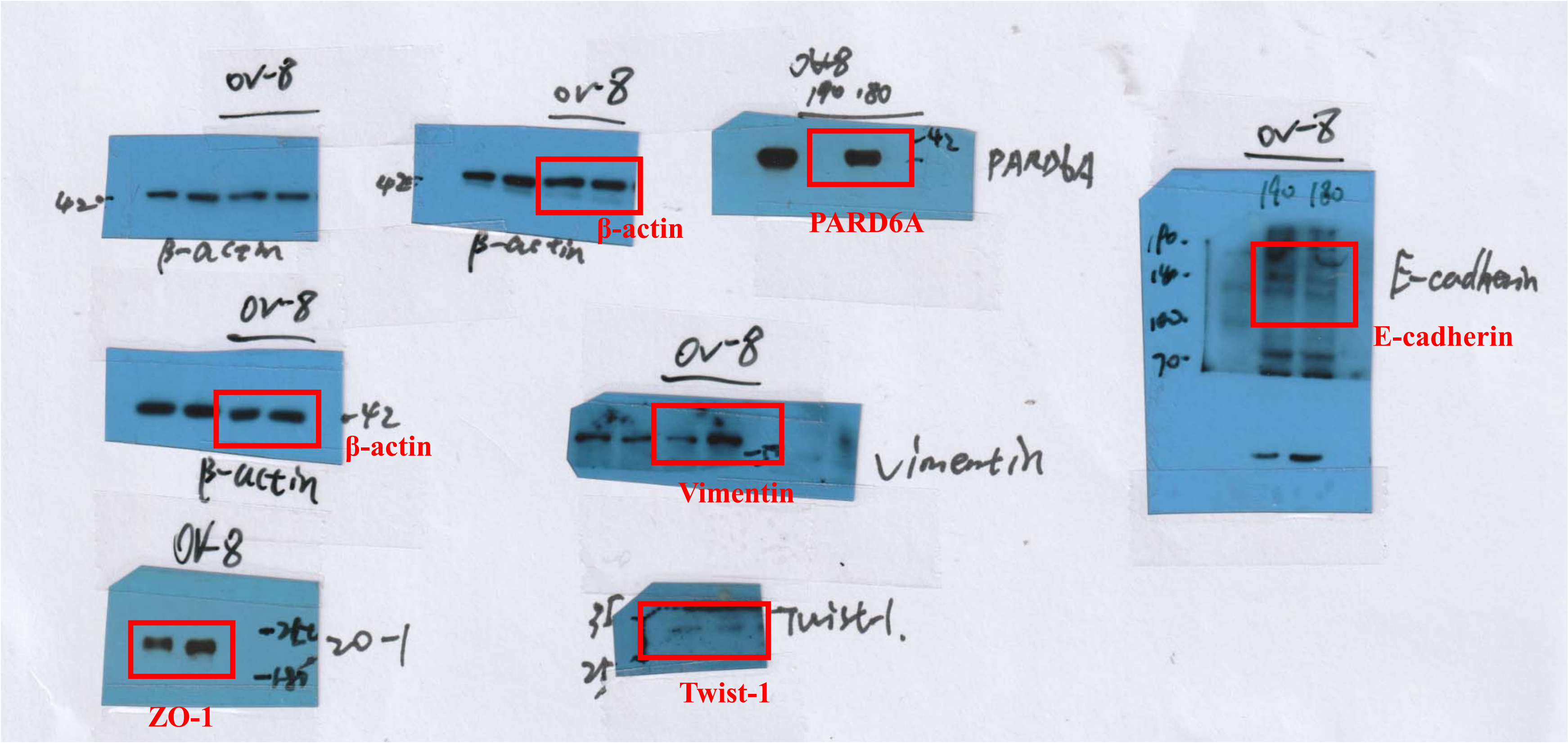

Figure 5A. SKOV3: SNAIL1

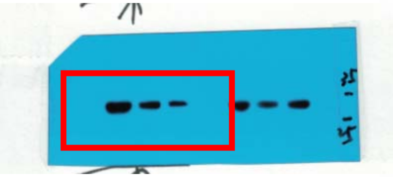

Figure 5B. A2780: SNAIL1

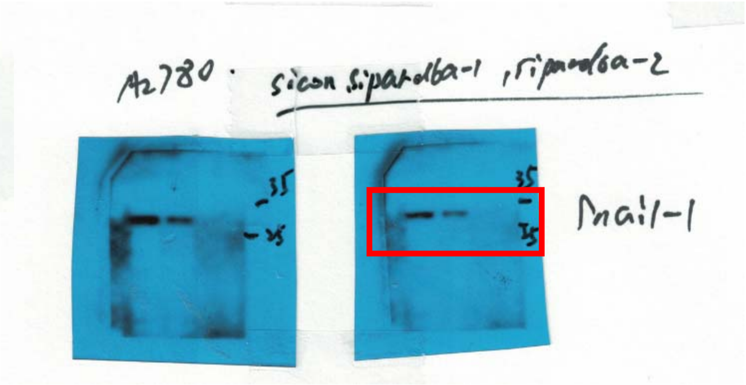

Figure 5C. HO8910: SNAIL1

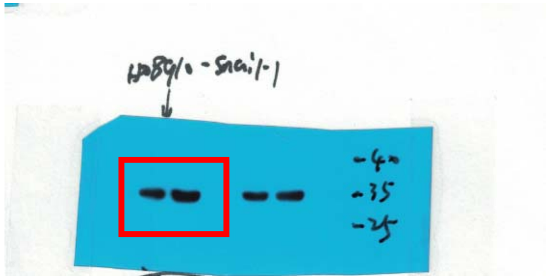

Figure 5D. OVCAR8: SNAIL1

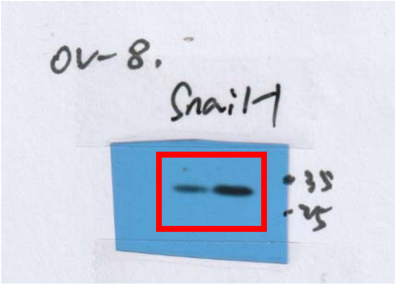

Figure 5A-D. SKOV3-A2780-HO8910-OVCAR8: Histon H3

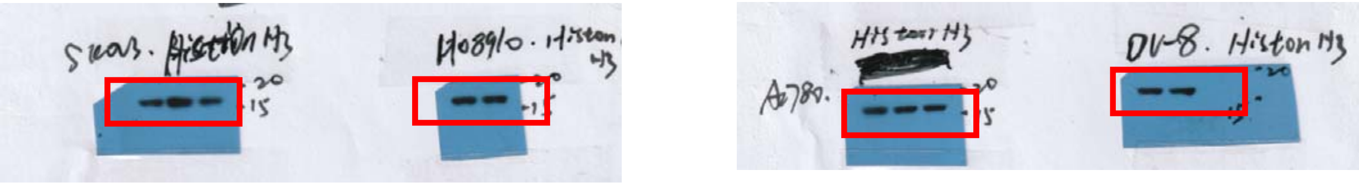

Figure 6C. SKOV3: PARD6A

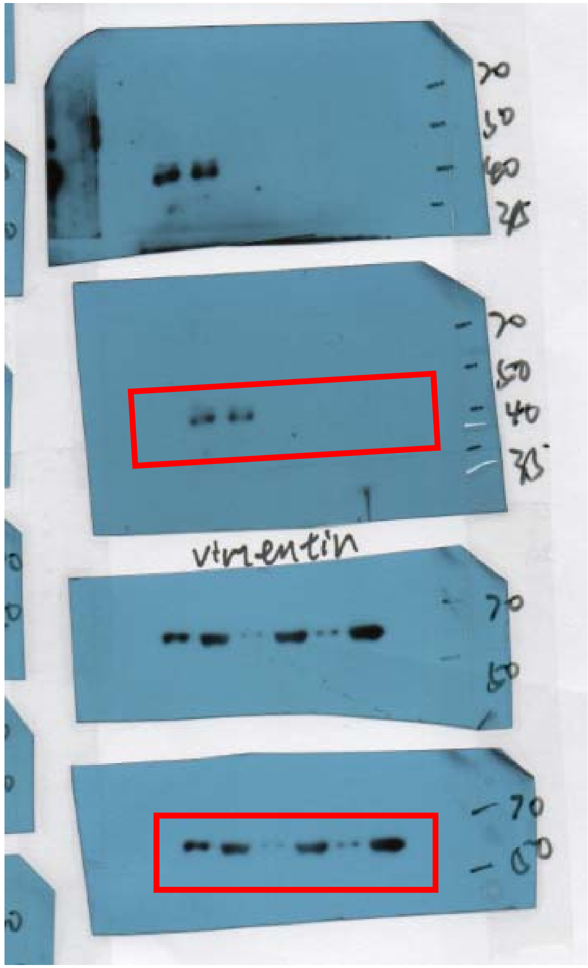

Figure 6C. SKOV3: Vimentin

Figure 6C. SKOV3: E-cadherin

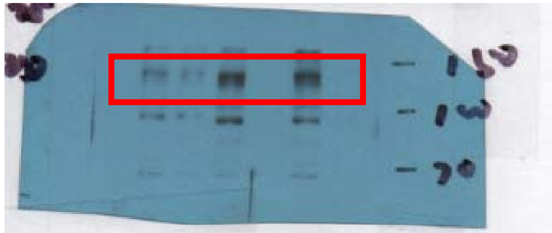

Figure 6C. SKOV3: β-actin

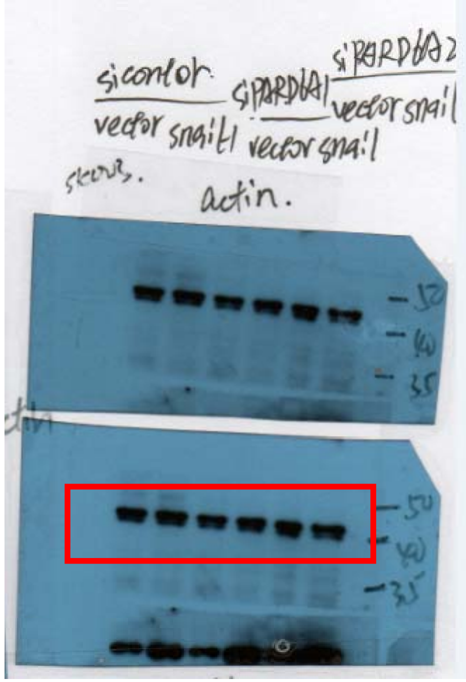

Figure 6C. SKOV3: Histon H3

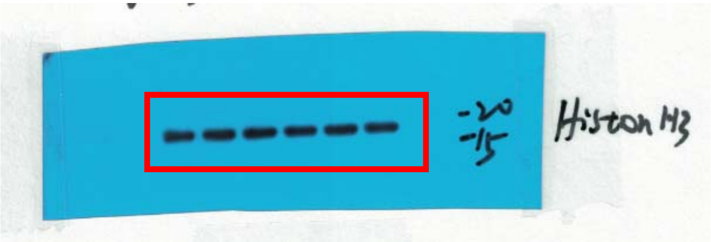

Figure 6C. SKOV3: SNAIL1

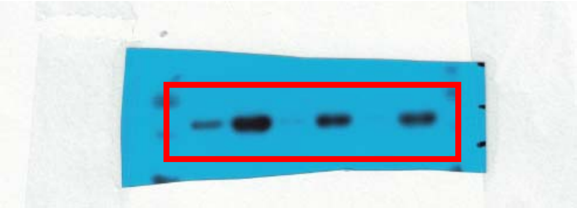

Figure 6F. HO8910: PARD6A

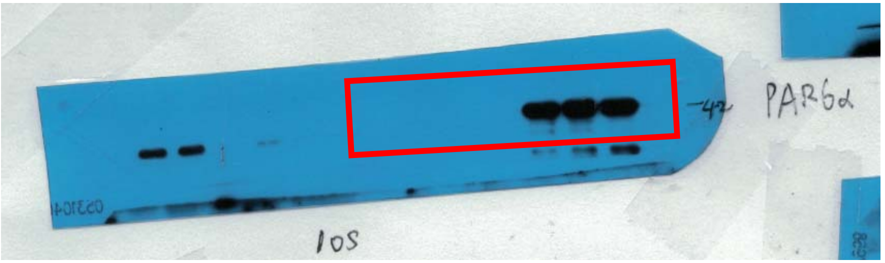

Figure 6F. HO8910: β-actin

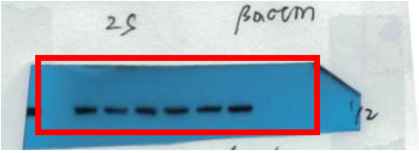

Figure 6F. HO8910: E-cadherin

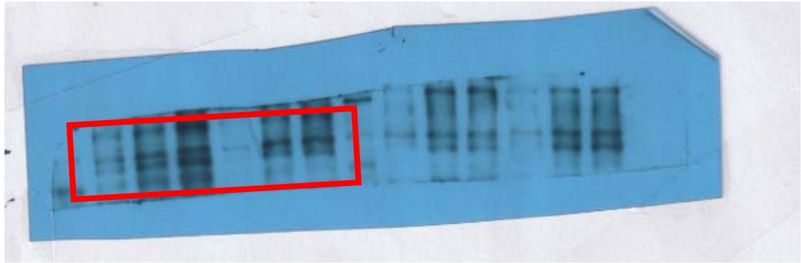

Figure 6F. HO8910: Vimentin

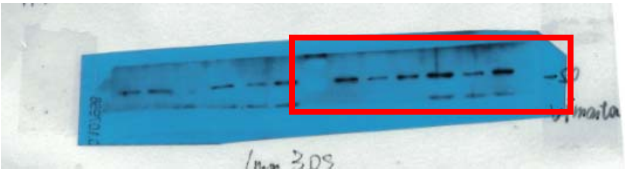

Figure 6F. HO8910: Histon H3

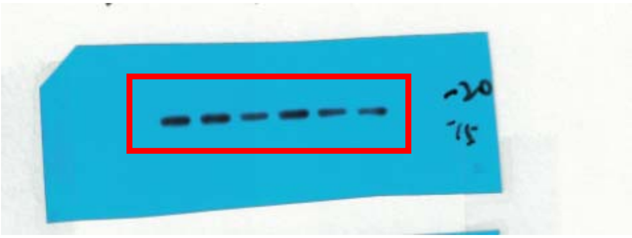

Figure 6F. HO8910: SNAIL1

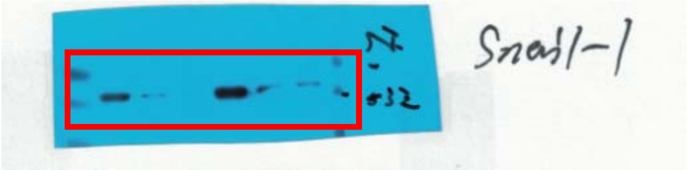

Figure 7A.

Figure 7A.SKOV3:RhoA

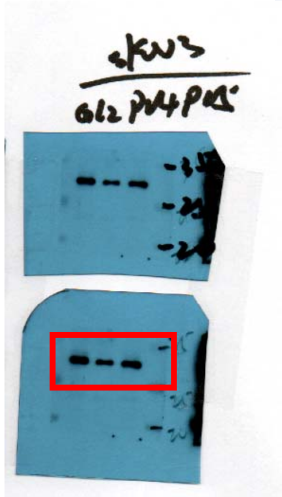

Figure 7A. SKOV3:β-actin

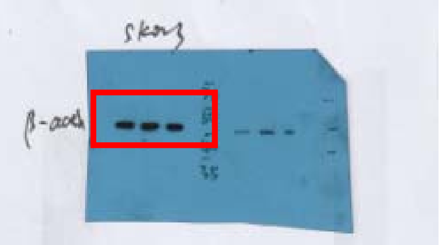

Figure 7A. A2780:RhoA

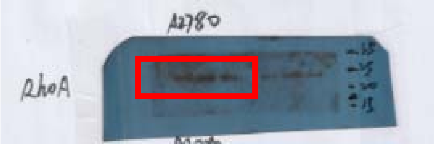

Figure 7A. A2780:β-actin

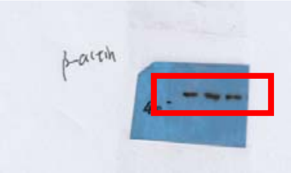

Figure 7A.OVCAR8:β-actin

Figure 7A.HO8910:β-actin

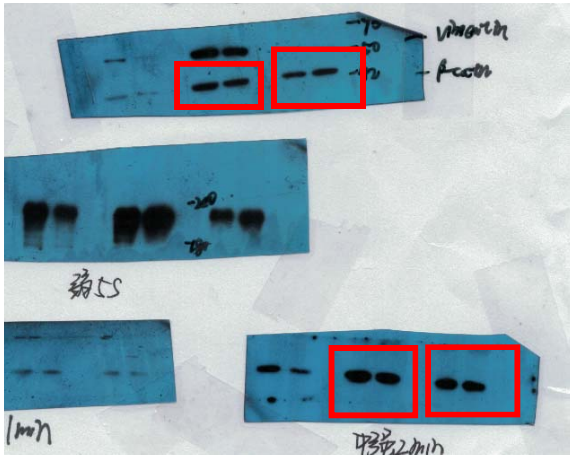

Figure 7A.OVCAR8:RhoA

Figure 7A.HO8910:RhoA

Figure 7C.

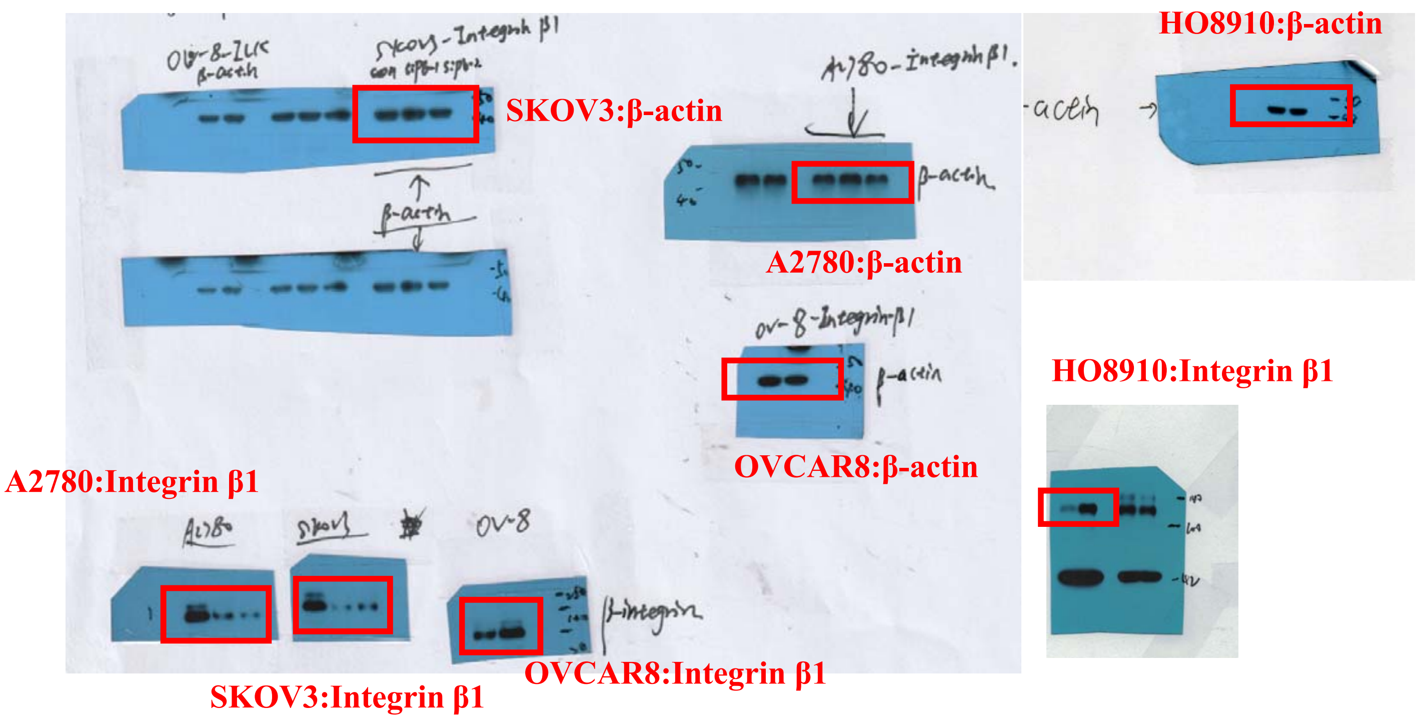
SKOV3:β-actin
HO8910:β-actin
A2780:β-actin
HO8910:Integrin β1
OVCAR8:β-actin
A2780:Integrin β1
OVCAR8:Integrin β1
SKOV3:Integrin β1

Figure 7D.

SKOV3:β-actin A2780:β-actin HO8910:β-actin OVCAR8:β-actin

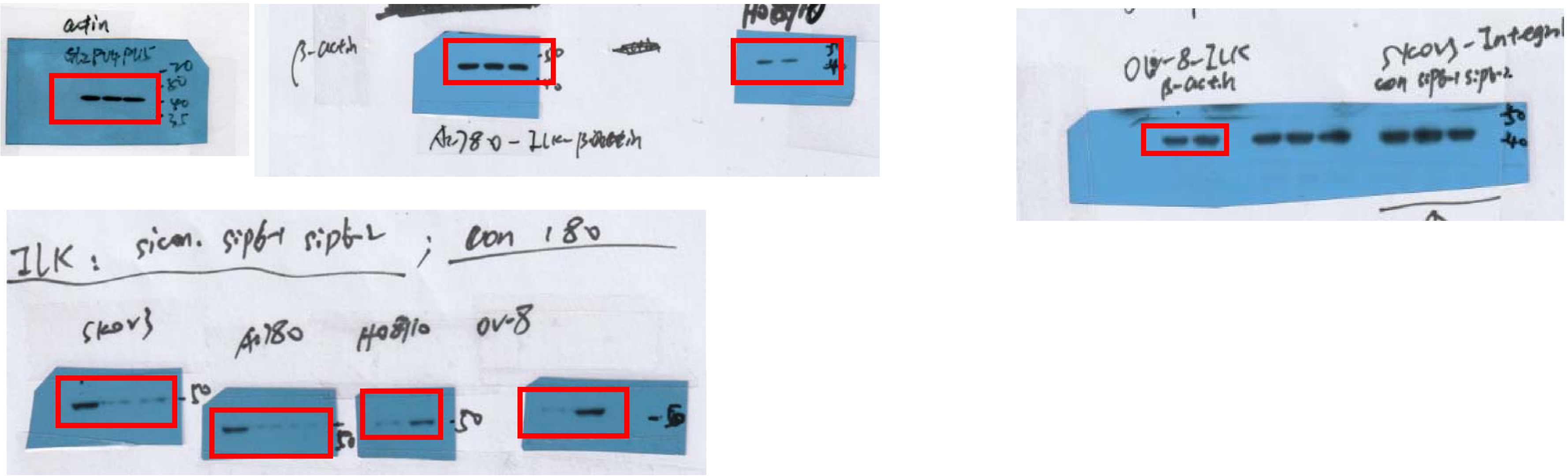

SKOV3:ILK A2780:ILK HO8910:ILK OVCAR8:ILK

Figure 7D. pGSK-3β

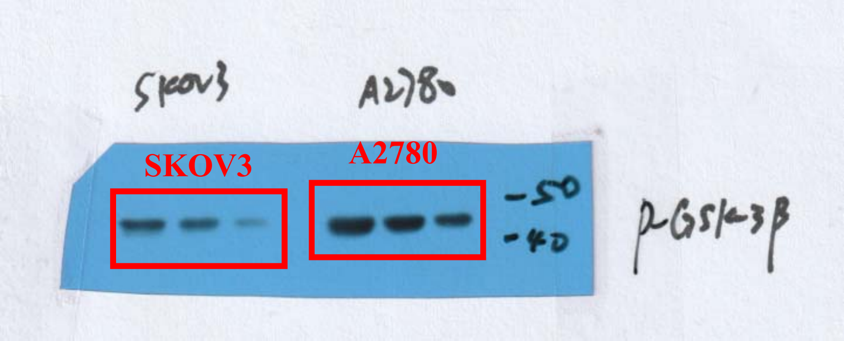

HO8910

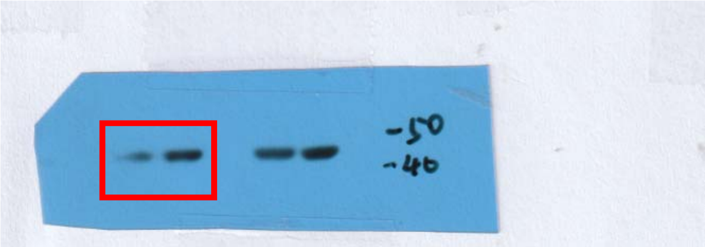

OVCAR8

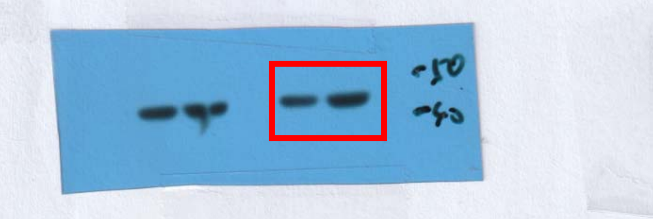

Figure 7D. GSK-3β

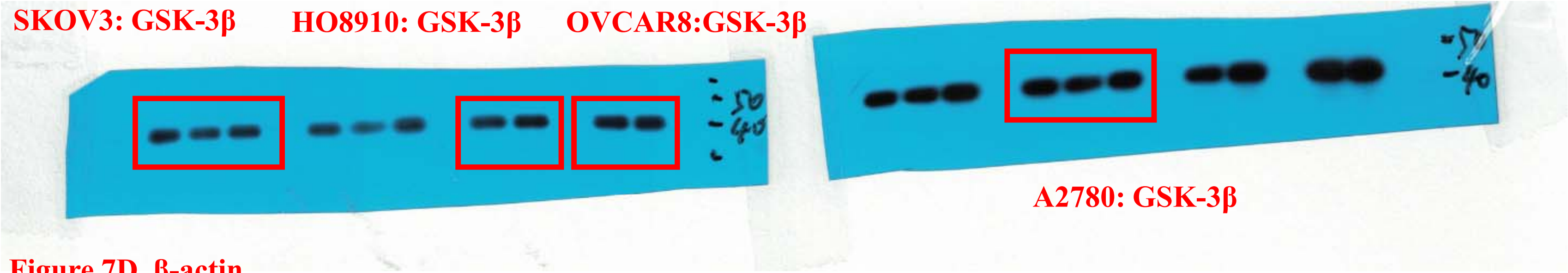

Figure 7D. β-actin

SKOV3: β-actin

A2780: β-actin

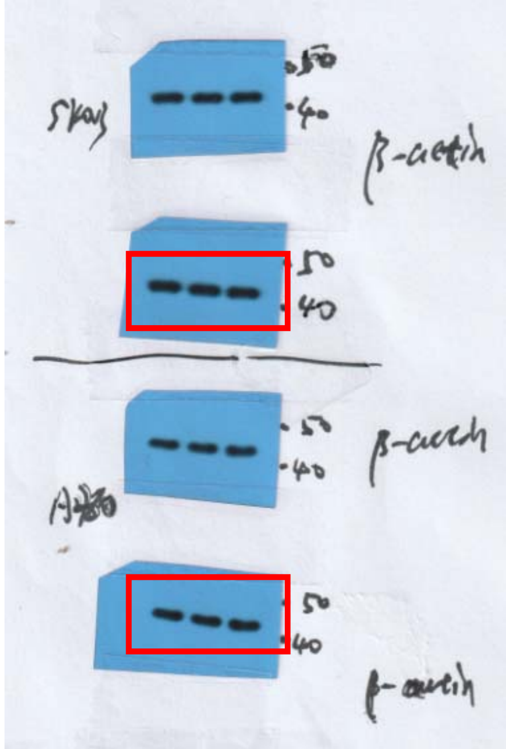

HO8910:β-actin

OVCAR8: β-actin

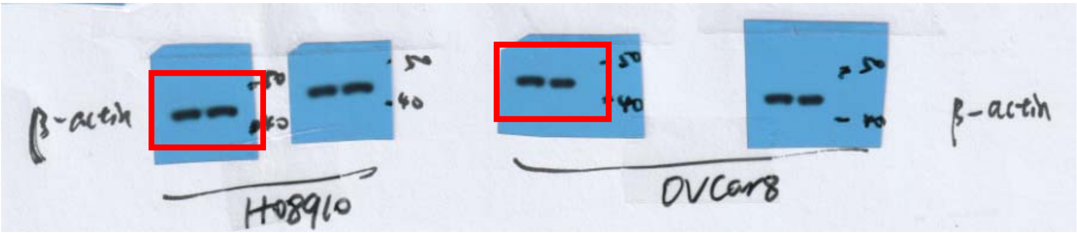

Figure 7F. SKOV3-A2780: ILK

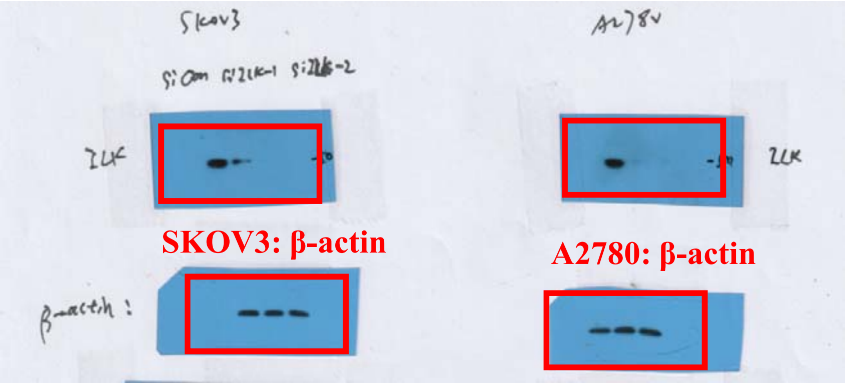

Figure 7F. HO8910-OVCAR8: ILK

HO8910:β-actin

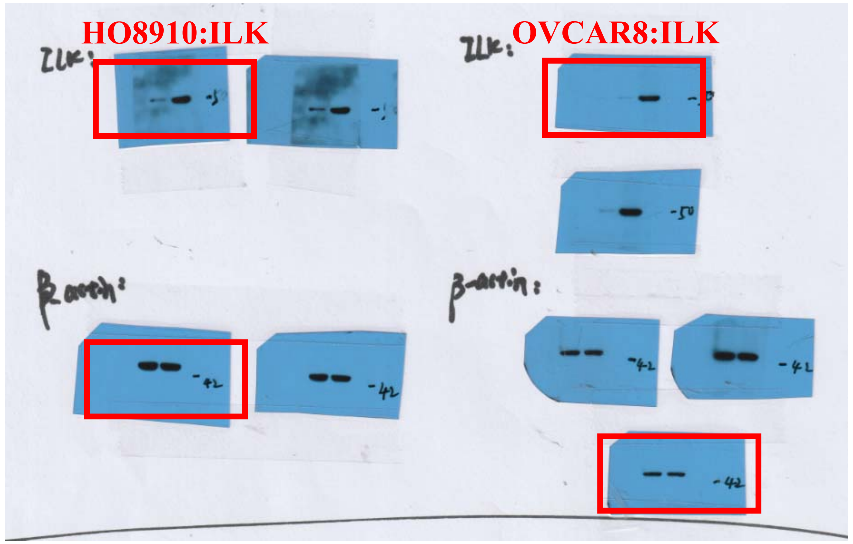

OVCAR8:β-actin

Figure 7F. SKOV3-A2780-HO8910-OVCAR8: pGSK3β

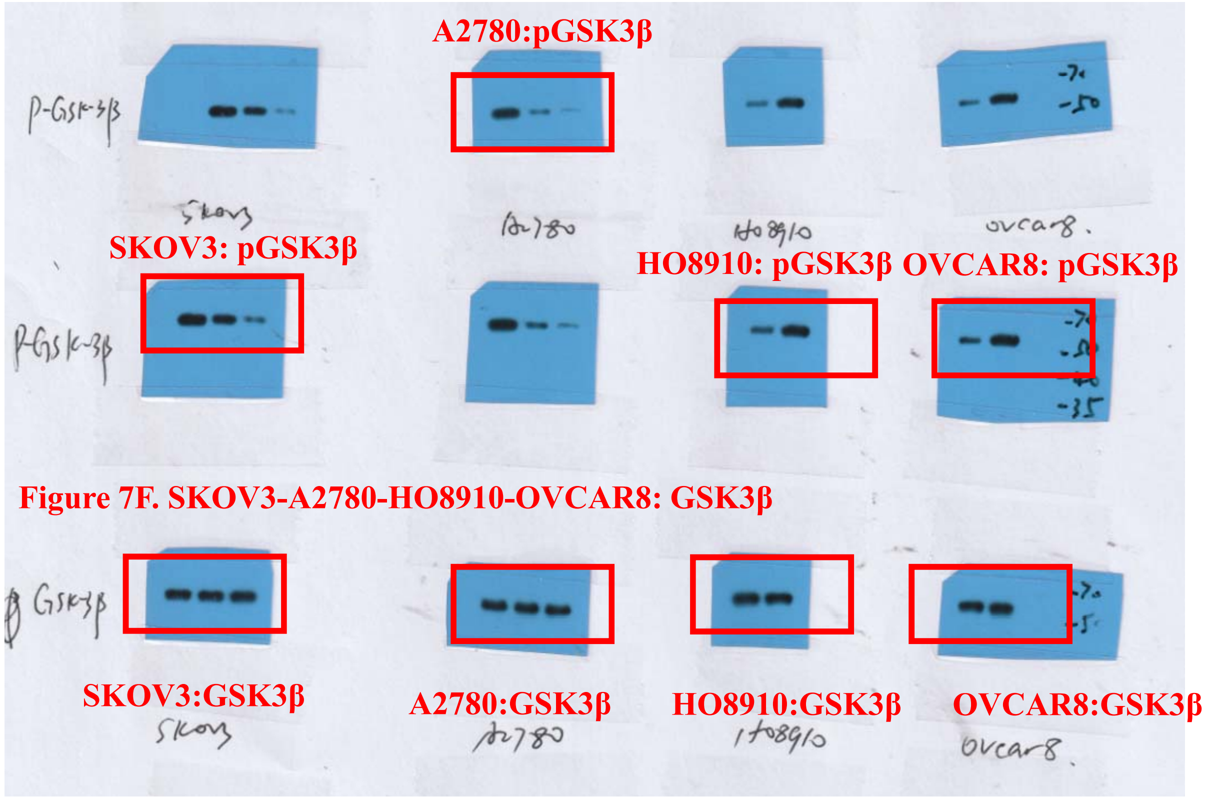

Figure 7F. SKOV3-A2780:SNAIL1

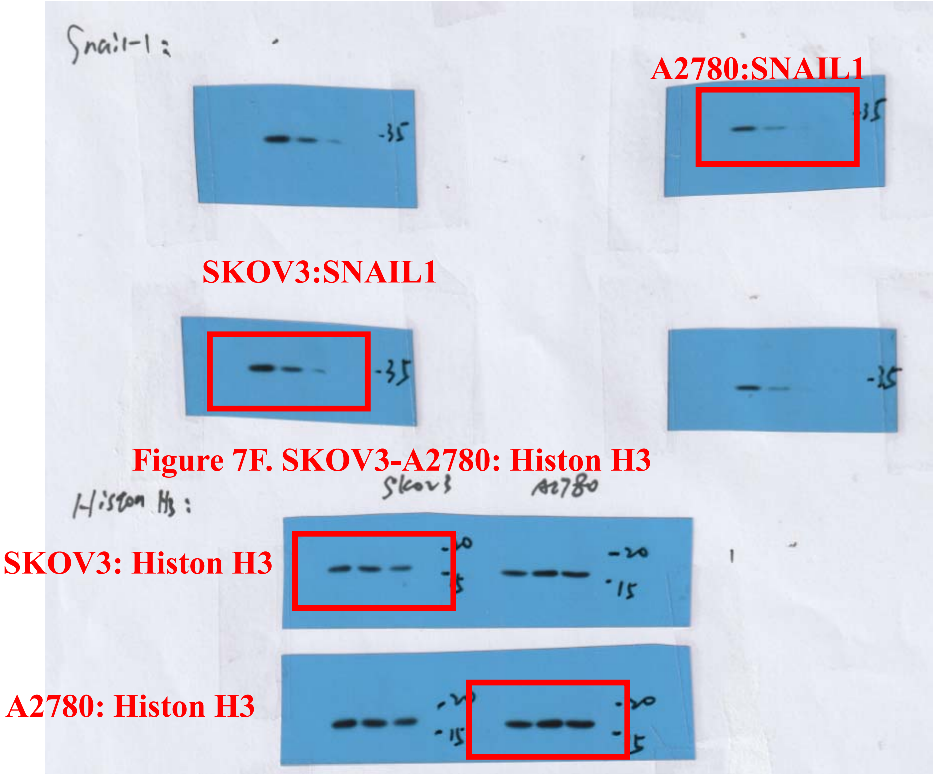

Figure 7F. HO8910-OVCAR8:SNAIL1

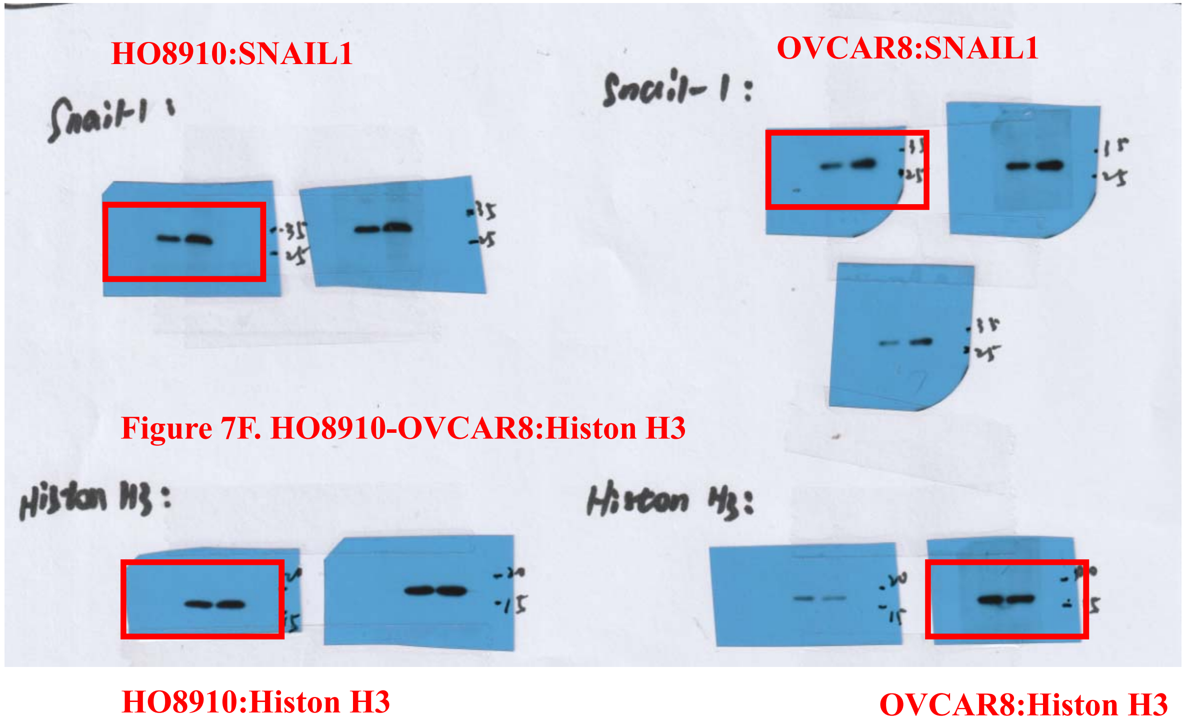

Figure S1C. SKOV3: PARD6A

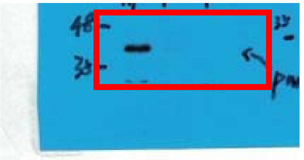

Figure S1C. SKOV3: β-actin

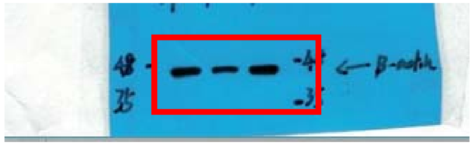

Figure S1D. A2780: β-actin

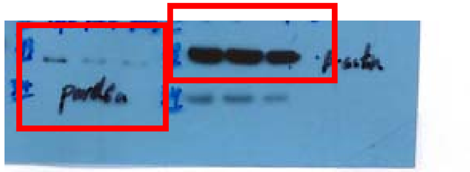

Figure S1D. A2780: PARD6A

Figure S3. β-actin

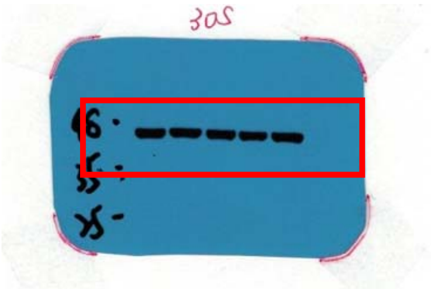

Figure S3. PARD6A

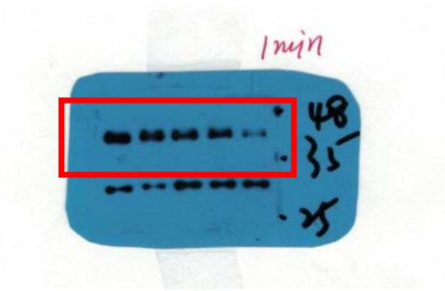

Figure S4. A2780: PARD6A

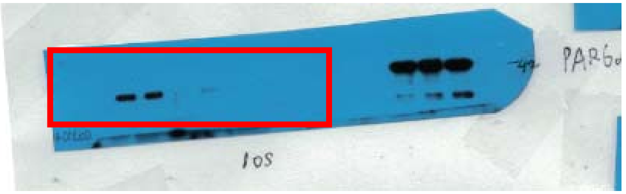

Figure S4. A2780: β-actin

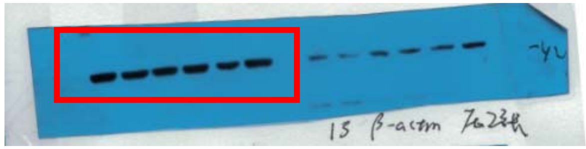

Figure S4. A2780: E-cadherin

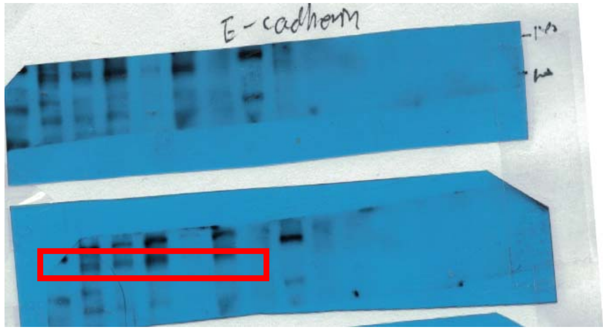

Figure S4. A2780: Vimentin

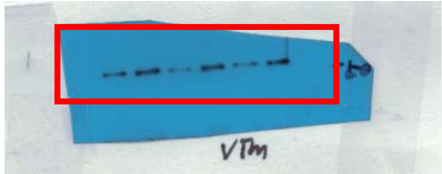

Figure S4. A2780: SNAIL1

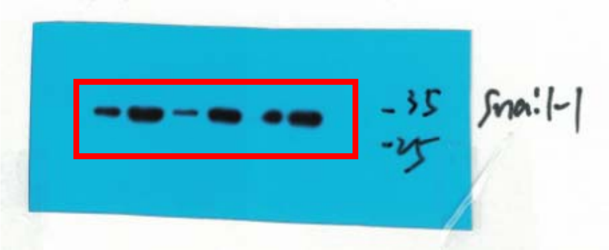

Figure S4. A2780: Histon H3

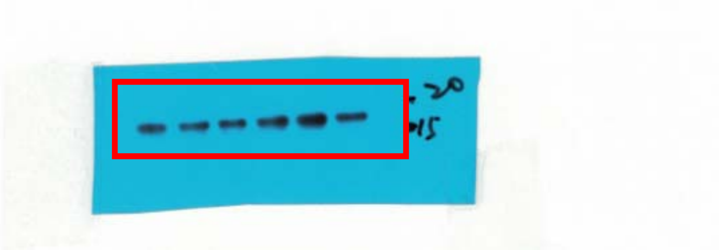

Supplement: Supplementary file 7 — Supplementary materials-original western blots [file 41419_2022_4756_MOESM7_ESM.pdf]
